# Comparison of Chemometric Problems in Food Analysis using Non-Linear Methods

**DOI:** 10.3390/molecules25133025

**Published:** 2020-07-02

**Authors:** Werickson Fortunato de Carvalho Rocha, Charles Bezerra do Prado, Niksa Blonder

**Affiliations:** 1 National Institute of Metrology, Quality and Technology (INMETRO), Av. N. S. das Graças, 50, Xerém, Duque de Caxias 25250-020, RJ, Brazil; wfrocha@inmetro.gov.br (W.F.C.R.); cbprado@inmetro.gov.br (C.B.d.P.); 2 National Institute of Standards and Technology (NIST), 100 Bureau Drive, Stop 8390 Gaithersburg, MD 20899, USA

**Keywords:** food analysis, chemometrics, non-linear methods, artificial neural networks (ANN), self-organizing maps (SOM), support vector machine (SVM)

## Abstract

Food analysis is a challenging analytical problem, often addressed using sophisticated laboratory methods that produce large data sets. Linear and non-linear multivariate methods can be used to process these types of datasets and to answer questions such as whether product origin is accurately labeled or whether a product is safe to eat. In this review, we present the application of non-linear methods such as artificial neural networks, support vector machines, self-organizing maps, and multi-layer artificial neural networks in the field of chemometrics related to food analysis. We discuss criteria to determine when non-linear methods are better suited for use instead of traditional methods. The principles of algorithms are described, and examples are presented for solving the problems of exploratory analysis, classification, and prediction.

## 1. Introduction

According to the Food and Agriculture Organization of the United Nations (FAO) [1], food safety refers to handling, preparing, and storing food in a way to best reduce the risk of individuals becoming sick from foodborne illnesses. This practice is very important for countries that export their products, and as such, food safety is part of regulations in many countries [2]. In both developed and developing countries, government institutions are responsible for the inspection of products, i.e., protecting the public’s health by ensuring the safety of food. Examples of these institutions include the Brazilian Health Regulation Agency (ANVISA) and the Ministry of Agriculture, Livestock, and Food Supply (MAPA) in Brazil, while the United States have the United States Department of Agriculture (USDA) and United States Food and Drug Administration (USFDA). These government bodies are tasked with enforcing required standards of nutritious food, animal feed, animal health, plant protection, clear information on the product origin, and content/labelling of food and various food related products [3,4,5]. Major problems that have been identified by these departments are related to adulteration and food frauds. To detect and quantify these crimes, laboratories have been using classical and instrumental methods for identification and quantification of chemical compounds. Modern instrumentation can generate complex data by spectroscopic, microscopic, and chromatographic methods that can be used to gain a better understanding of food safety. However, extracting essential information from these data in their raw form often is too complex for the human brain to process.

Multivariate methods can help extract relevant qualitative or quantitative information from complex data, and these methods can be used in food analysis. The use of non-linear methods is becoming commonplace for researchers building models for classification, pattern recognition, optimization, and prediction. The advantage of non-linear methods can be highlighted by their capacity to handle datasets that exhibit the following characteristics [6]:

- non-linearity, allowing a better fit for the data;
- noise insensitivity, providing accurate prediction in the presence of data uncertainty and measurement errors;
- high parallelism, implying fast processing and hardware failure tolerance;
- generalization, enabling application of the model to unknown data.

Non-linear methods are also not affected by limitations of Beer–Lambert law [7] that occur with analytical instruments and chemicals, such as changes in refractive index at high analyte concentration, shifts in chemical equilibria as a function of concentration, scattering of light, fluorescence or phosphorescence of the sample, and nonlinear detector response [8]. Thus, the application of adequate multivariate methods for the analysis of complex datasets can solve demanding analytical problems in the field of food safety.

This review presents a retrospective of the studies carried out from 2008 to 2018 that make use of non-linear methods as a research tool in the field of food analysis. The goal of this review is to show how non-linear methods have solved problems of classification and prediction, as well as to discuss the advantages and disadvantages of these methods with respect to traditional multivariate techniques. We recognize a need for more widespread knowledge of application of non-linear methods and have attempted to help fill the vacuum with this review.

### 1.1. Artificial Intelligence and Machine Learning from a Chemometrics Perspective

**Figure 1:**
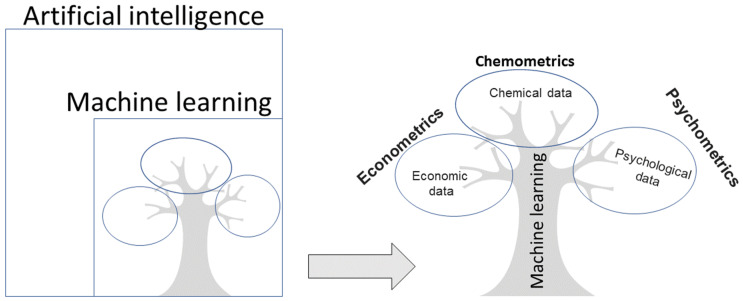
Machine learning (ML) is subset of Artificial intelligence (AI) and Chemometrics is machine learning used in chemistry.

Artificial intelligence research involves building computer programs designed to behave or mimic human brain functions such as talking, playing soccer, and planning. Within the field of artificial intelligence, a very widespread area known as machine learning has developed, which involves the applications of different algorithms that are able to learn and improve from experience. Machine learning is divided into two types: supervised and unsupervised [9]. In supervised learning, the goal is to predict the value of an output variable based on several input variables; in unsupervised learning, the goal is to describe the associations and patterns among a set of input variables without an output variable. Machine learning methods for data evaluation and interpretation can be used in many fields, and often have different names depending on the area of study. For example, when applying machine learning in psychology, the discipline is called psychometrics [10,11], in economics, econometrics [12], and in chemistry, chemometrics [13] (Figure 1).

The discipline of chemometrics can be performed by both linear and non-linear methods. Linear methods include principal component analysis (PCA), hierarchical cluster analysis (HCA), principal component regression (PCR), partial least squares regression (PLS), soft independent modeling of class analogy (SIMCA), linear discriminant analysis (LDA), and partial least squares discriminant analysis (PLS-DA). Non-linear methods include artificial neural networks (ANN), support vector machine (SVM), and self-organizing map (SOM). New non-linear methods are constantly being developed, and existing methods are constantly being modified. Herein, we briefly describe three different groups of non-linear methods (artificial neural networks, self-organized maps, and support vector machine) on which this review will focus. A detailed explanation of the theory and application of different types of machine learning algorithms in food testing is provided in the reference list.

#### 1.1.1. Artificial Neural Networks (ANN)

**Figure 2:**
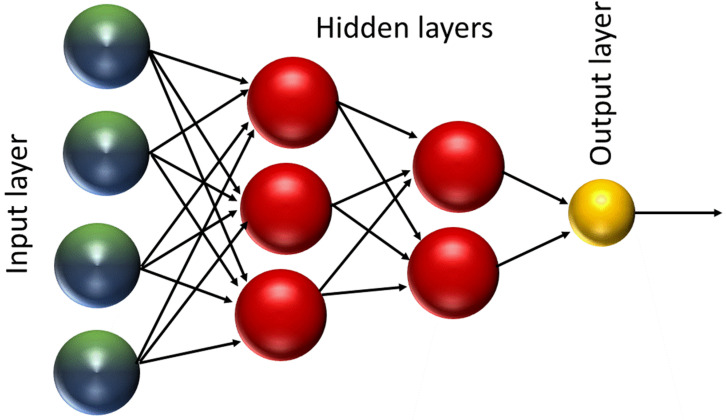
Multilayer perceptron showing input, hidden, and output layers and nodes with feedforward links.

An artificial neural network (ANN) is a non-linear computational model attempting to simulate human brain structure and decision making [14]. There are many types of neural network, such as the convolutional neural network (CNN), recursive neural network (RNN), and feed forward neural network (FFNN). The simplest form of ANNs is the FFNN, which consists of one or more hidden layers of perceptrons (neurons) (Figure 2) [15]. Each perceptron has an activation function which computes an output signal depending on the weighted input received. Perceptrons from one layer are connected to perceptrons in the next layer and the output signal flows from one layer to the next without any feedback connections [16,17]. The connection between the perceptrons is characterized by different variables. These are a weight and bias values associated with each node as well as the transfer function that determines the state of a node based on the weight and bias parameters [18]. FFNN requires supervised training by taking input of example data sets and desired output results that are fed to the network multiple times. Each time the weights of the activation function are adjusted so that the error in the output is minimized.

#### 1.1.2. Self-Organizing Maps (SOMs)

**Figure 3:**
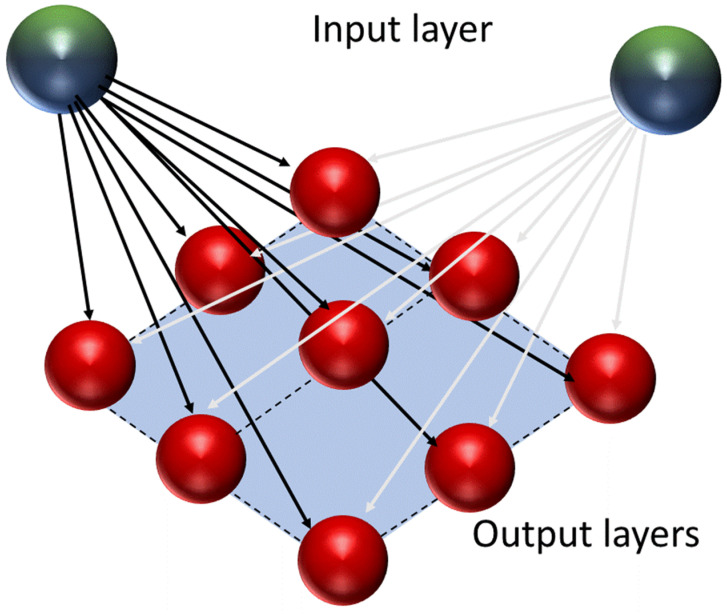
Schematic illustration of the structure of a SOM with two input neurons and 3 × 3 Kohonen neurons.

The concept of self-organizing maps (SOMs), sometimes referred to as Kohonen maps or Kohonen networks, was developed by Teuvo Kohonen. SOM networks are based on an unsupervised training algorithm that consists of input nodes and a grid of computational nodes (neurons) [19]. Each input node is connected to every computational node (Figure 3).

These neurons compete among themselves for activation as the one that most closely resembles the input vector. If the input data exhibits some similarity across the input classes, the neurons will organize themselves showing patterns of similarity in a grid. SOMs are used to transform large multi-dimensional datasets into a lower-dimensional display that better represents similarities within a dataset. SOM analysis requires several parameters to be specified by the user. The main parameters are number of nodes (SOM grid size), topology of the map, map shapes, initialization, and training algorithms. According to Tian et al. [20], it is possible to use the following equation to define the number of nodes:

M = 5√N where “N” is the number of samples in the dataset and “M” is the number of neurons. The topology of map can be quadrangular, rectangular, or hexagonal and map shapes can be planar, cylinder and toroid. There are different ways to do the initialization and training. Initialization can be done in a random or linear manner. Sequential or batch algorithms can be used during the training phase [21].

#### 1.1.3. Support Vector Machines (SVMs)

**Figure 4:**
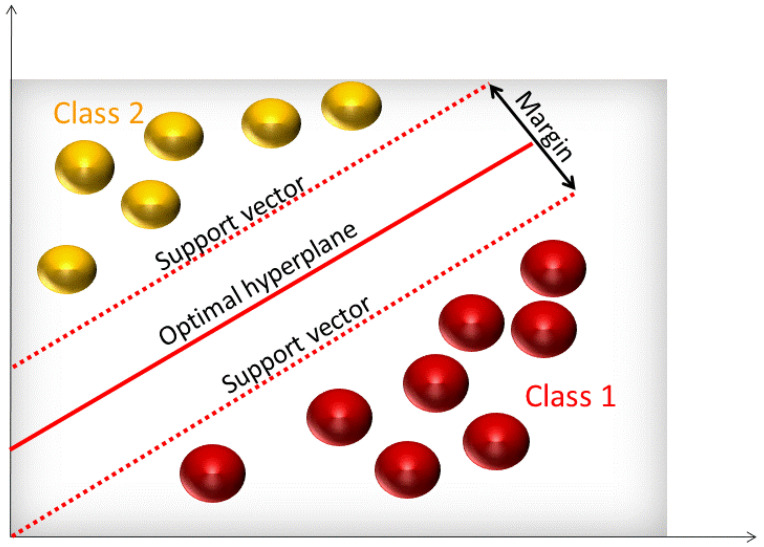
Representation of general classification hyperplane that maximizes the margin of the training data.

In classification problems, support vector machines (SVMs) are used in determining separation functions, while for prediction problems they can be used to carry out functional estimation. The output of an SVM is the best separating hyperplane that categorizes input data [22]. As seen in Figure 4, support vectors are data points closest to the hyperplane that separates the two classes.

Maximum margin is defined by doubling the minimum distance from support vector points to the hyperplane. Training SVMs requires supervised learning that uses an iterative training algorithm to minimize the error of the output. To build a good SVM model with low error rate, a proper kernel function must be selected along with the optimal kernel parameters. There are many kernel functions that can be taken into consideration such as linear, quadratic, and radial basis functions. The most common one is the radial basis function (RBF). This function requires two parameters: gamma and cost. The gamma parameter controls the shape of the separating hyperplane [23] while the cost factor allows for a tradeoff between calibration error and model complexity [24].

### 1.2. Input Data

**Figure 5:**
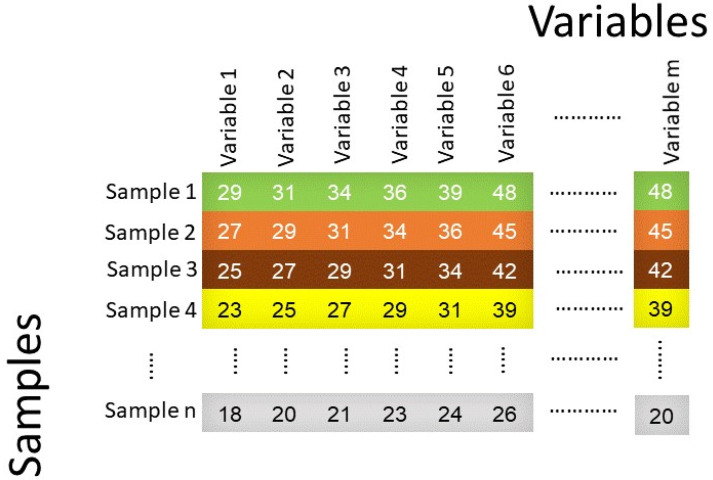
Representation of data in matrix form.

Non-linear models can be built from simple multi-element analysis such as chromatography, spectroscopy, mass spectrometry, thermal analysis, electrochemical analysis, microscopic and diffraction scattering techniques to study food analysis. These data are represented as a matrix consisting of rows and columns, where the rows represent samples and the columns represent variables (Figure 5).

The variables may represent the number of chromatographic peaks, biological measurements, or spectroscopic measurements. The variables depend on the instrumentation used for data acquisition and can be from more than one instrument type. Chemometric methods such as PCA are commonly used to reduce a dataset before applying non-linear methods, which helps with selection of important variables to measure and improve accuracy of the model, reduce overfitting, and decrease training time.

### 1.3. How to Test Whether Dataset is Linear or Non-Linear?

While literature recommendations can help with determining the linearity of a dataset, no official guideline exists for selecting between linear and nonlinear methods to fit these datasets. Regression analysis is used to determine whether a relationship between two or more variables can be represented by a straight line with small residuals (errors) exhibiting random behavior. Many statistical tests can be used for making quantitative and qualitative decisions about residuals from a regression analysis, such as the Durbin–Watson test [25], Breusch–Pagan test [26], Goldfeld–Quandt test [27], Shapiro–Wilk test [28], Kolmogorov–Smirnov test [29], and residual plots [30,31]. If adequate fit cannot be obtained using a linear method, the relationship between input and output data is deemed not linear, and non-linear regression can be used.

In classification problems, however, a linear relationship between input and output data is less important than confirming whether data can be separated by a linear classifier. Generally, food analysis data can be separated by linear classifiers such as PLS-DA, SIMCA, and LDA. In some cases, classes may not be separable by a linear boundary used by these models and non-linear methods are recommended for capturing non-linear patterns of the dataset. Methods, such as SOM, SVMs, and FFNN, are particularly suitable for modelling non-linear boundaries between samples belonging to different groups.

Therefore, to determine whether certain data is non-linear and whether a non-linear model can be used for modeling, the following actions are a good guide for making the decision [32,33,34]:

- make a histogram graph of the raw data;
- create probability plots to identify the data distribution;
- perform distribution tests to identify the distribution probability that the data follows;
- check the goodness of fit test results for the distribution tests.

If the error and goodness of fit test results show high accuracy and the relationship among variables appears as a straight line, then it implies that the dataset is linear in nature.

After applying these steps, along with the specific knowledge of the area being studied and the behavior of the data, it is possible to determine whether the nonlinear model is more appropriate than the linear one for a study.

### 1.4. Identifying Food Analysis with Non-Linear Methods

**Figure 6:**
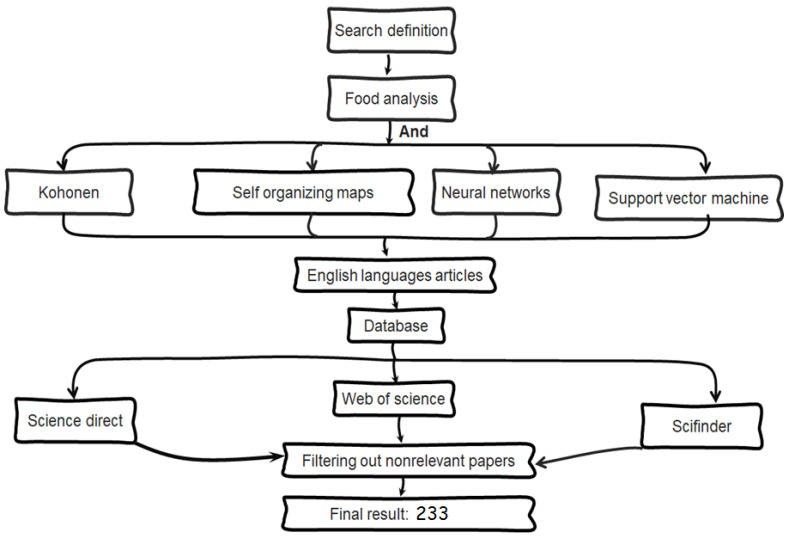
Method utilized for the search and exclusion of papers.

References used for this review were obtained by in-depth search of three distinct online accessible databases: Science Direct, SciFinder, and Web of Science. Although each database engine has a different interface for performing corresponding searches, the main search term “food analysis” was used in combination with terms for different types of machine learning techniques; namely, “Kohonen”, “self-organizing maps”, “neural networks”, and “support vector machine”. Research papers that did not relate to our search topic were discarded as well as papers not written in English. Review papers were also discarded order to avoid circular referencing. These criteria produced a list of a total of 233 references used for this review (Figure 6).

The references were then divided into two groups for studying classification and prediction problems. Using food grouping posted by National Institutes of Health as a guideline [35], the foods were organized into seven different groups: grains, vegetables, fruits, protein, dairy, oils, and others (e.g., alcohol, spices, added sugars).

## 2. Application of Non-Linear Methods on Food Groups

Food analysis problems can be grouped into prediction and classification problems. A classification problem is when a sample is assigned to one group from a set of possible groups (classes) based on a series of experimentally measured indices. In classification problems, the output variables are usually binary categories, such as “good” or “bad”. On the other hand, in a prediction problem the output variable is a quantity express by a number, such as “2.08” or “0.01” and “8”. Regression analysis is one of the non-linear methods used to study prediction problems. According to Wold et al. [36], regression analysis is statistical method for estimating the relationship between two or more variables of interest. In chemometrics, non-linear regression methods have been used by many authors to examine the influence of one or more independent variables on a dependent variable. In the field of food analysis, the independent variables are represented by measured data obtained from different analytical techniques while dependent variables are represented by the property of interest, e.g., sugar content, concentration of herbicide, classification of geographical origin of food, quantification of microbial spoilage, additives, pH, firmness, and soluble solids in foods. Non-linear models such as neural networks can be used for prediction or classification.

### 2.1. Classification

Most of the papers reported their findings as a measure of accuracy, which in the context of classification is defined as the percentage of the correctly classified data points within a dataset. However, a high accuracy rate does not necessarily imply a good classification model [37,38,39]. In this accuracy paradox, some models with lower accuracy may have better predictive ability compared to models with higher accuracy. Specifically, this can occur when training data set is not balanced, where one class of data represents large majority of the training input. Accuracy of a classification model can also be reported as misclassification rate. There are two types of misclassification: Type I when a model identifies a point as not belonging to a class A when it actually does belong to class A (also known as false negative) and Type II when a model identifies a point to belong to class A when it actually does not belong to class A (also known as false positive) [40]. Overall accuracy describes the average of true positive rate and true negative rate [41,42]. In the context of classification, prediction refers to the use of a classifier model for determining the class that an unlabeled object likely belongs to [43].

Sensitivity, specificity, efficiency and correct classification rate (CCR) are terms used to describe the performance of class modeling techniques. Sensitivity describes the fraction of correctly identified objects from a modeled class, while specificity describes the ability to accurately detect/reject objects from the other classes. Efficiency of the model, in this scenario, represents the geometric mean of the sensitivity and specificity [44]. CCR represents a ratio of correctly classified samples to total number of samples in the data set used during the testing or cross validation of a classifier model [45].

#### 2.1.1. Vegetables

Visible and short-wave near-infrared (Vis/SW NIR) diffuse reflectance spectroscopy is a non-destructive and fast technique that can be used for gathering sample data about food products. One use is classification of tomatoes by different genotypes. Using LS-SVM, Xie et al. [46] achieved a 100% classification accuracy when using the whole spectral region. While a 100% correct classification can also be obtained using a discriminant analysis method, LS-SVM performed faster with the dataset made up of greater varieties of tomatoes. When selecting only the most relevant wavelengths, overall classification decreased to 96.8% but was deemed as an acceptable classification accuracy.

The electronic nose (E-nose) is an instrument designed to recognize samples by olfaction mimicking the way humans sense smell [47]. It can be used to classify freshly squeezed tomato juice based on different storage times of the tomatoes for tracing product quality. Hong et al. [48] showed that BPNN outperformed SVM with validation set accuracies of 97.0% for BPNN and 94.2% for SVM. They also showed that a semi-supervised Cluster-then-Label approach based on spectral clustering can provide classification accuracy of 98.7%.

The quality control of potato chips can be complex due to oil residues, various additives, and seasonings. Using NIR spectroscopy, Ni et al. [49] have established that LS-SVM model was able to clearly predict four parameters (fat, moisture, acid, and peroxide values of the extracted oil) for qualitative and quantitative measurements. Comparing different methods for classification of potatoes based on sugar levels showed that ANN did not perform as well as linear methods such as LDA and PLS-DA. These results suggest that an improvement in classification accuracy could be accomplished by increasing the number of samples and using SVM [50].

Identification of contamination by food borne pathogens in packaged vegetables is important for food quality control. Escherichia coli (E. coli) was taken as the target microorganism and E-Nose was used for analysis of volatile metabolites from the headspace of packaged alfalfa sprouts. Data generated by the E-Nose sensor was then successfully classified using SOM algorithm, showing different subgroups with different number of E. coli [51]. The limitation of E-Nose method, however, is the requirement of E. coli counts higher than 105 colony-forming units per gram (CFU/g).

A classification model for traceability of geographical origin of Boletus edulis known as “porcini mushrooms” was investigated by Li et Al. [52]. Mushrooms were collected from nine regions of Yunnan Province in China. Mid-level fusion (a method that utilizes feature extraction or variable selection prior to multivariate analysis [53]) was performed on data from FT-MIR spectroscopy and thirteen elements determined by inductively coupled plasma-atomic emission spectrometry (ICP-AES). Thus, thirteen subsets were generated for data analysis. Grid search (GS) and genetic algorithm (GA) techniques were used for the optimization of the radial basis function used in the SVM model. Classification accuracies obtained, for both GS-SVM and GA-SVM, were 81.4% for calibration and 90.9% for validation datasets. Yao et Al. [54] obtained even better classification accuracies of 99.1% for training and 100% for test sets, using SVM to classify data from FT-IR and ultraviolet-visible absorption (UV-vis) spectroscopies coupled with data fusion. Fu et al. [42] investigated use of NIR coupled with interval-combination one-versus-one least squares support vector machine (IC-OVO-LS-SVM) for classifying Chinese *Ganoderma lucidum* mushroom by origin. Total classification accuracy reported by this method was 93.2%, while average sensitivity and specificity were 93.1% and 99.7%. This indicates that NIR can potentially be used with machine learning algorithms for classification in food industry.

Multilayer perceptron artificial neural networks (MLP-ANNs) were applied to data generated by inductively coupled plasma optical emission spectrometry (ICP-OES) for classification of geographical origin of Spanish paprika. Samples from La Vera (Extremadura) and Murcia origins were analyzed and classification accuracy of 99 ± 2% was reported with the MLP-ANN technique [55].

Postharvest physiological deterioration (PPD) is one of the major problems in quality of cassava roots which are used for human consumption as well as animal feed. Several genotypes of cassava roots were screened for chemical and enzymatic composition during PPD. The classification of fresh samples and those at stage 11 of PPD was performed by Urraota et al. [56] using various methods such as ANN, K-nearest neighbors (KNN), and SVM. Results showed that SVM method with radial kernel had the best classification accuracy compared against other chemometric methods.

Table 1 summarizes results from the articles describing chemometric applications along with statistical parameters used to compare the different methods and applications for the study of geographical origin and quality control of vegetables.

#### 2.1.2. Fruits

Discriminating red bayberries on presence of bruises is used for food quality assurance, consistency, and consumer confidence. Food images were captured by a digital camera and fractal analysis software used to determine fractal parameters while a color histogram tool was used to capture RGB intensity values from color images. PCA was used for converting fractal spectral data to a lower dimensionality. Using SVM to process the data, classification accuracy of 100% was reported for fractal parameters while 85.3% was reported for RGB intensity values [57].

Characterizing and detecting the non-visible mechanical damage of blueberries with time evolution can help to discard damaged berries, leading to packages of higher quality that can be stored for up to one year in a freezer [58]. Reflectance, transmittance, and interactance imaging spectroscopy were used to generate samples from 737 blueberries. Using multi-layer perceptron with back propagation ANN, classification accuracy of 77.8% was obtained with reflectance spectroscopy, while 100% accuracy was obtained with transmittance spectroscopy. The study also showed that, except for the first 12 h after the impact, good blueberries were easier to classify than damaged ones. In the first 12 h after the impact, classification accuracy of good blueberries was 56.3%, while classification accuracy of damaged blueberries was 88.4%. Accuracies for classifying blueberries 1 day and 2 days after the impact were 95.2% and 92.1% for good berries, while classification of damaged blueberries was lower at 55.8% and 74.4%.

Electronic Tongue (E-Tongue) data processing has been used for discrimination between 100% and 10% orange juice. Each class of orange juice consisted of 108 samples for a total of 216 analyses. Comparison of random forest (RF) classification against two non-linear techniques, BPNN and SVM, showed that all three techniques gave the same prediction accuracy of 100% [41]. A study conducted by Qiu et al. [59] showed that data from E-Tongue delivered a higher accuracy in classification of processed strawberry juices compared to E-Nose. However, grouping the two methods together delivered 100% accuracy with RF or SVM algorithms. The study also showed RF having slight edge over SVM when using E-Nose datasets.

Bunch withering disorder is one of the greatest problems facing the production of Mazafati variety of date fruits in Iran. Because no visual signs of the withering disease exist at the onset of infection, NIR spectroscopy was used as a nondestructive method for discrimination between healthy and diseased dates [60]. Three different methods were used on data samples to differentiate between healthy and diseased dates. Classification accuracies reported for these methods were 82% for SIMCA, 93% for PLS-DA, and 86% PCA-ANN.

Geographical origin is known to have a great impact on the quality of chayote fruit, *Sechium edule (S. edule*). A study was conducted to investigate how mineral composition of the fruit could be used as a discriminating factor to determine geographical origin of *S. edule* in Argentina. After microwave digestion, major and trace element composition was determined using ICP-OES [61]. LDA, KNN, PLS-DA, and SVM were applied for classification of a 92-sample data set. Discrimination accuracy results obtained for each of the methods were 89.1% for LDA, 84.7% for KNN, 82% for PLS-DA, and 87% for SVM, showing that LDA displayed the highest ability for predicting the geographical origin of the samples. In a separate study, SVM, LDA, KNN, PLS-DA, and RF were compared for prediction of the origin of lemon juice from 4 different Argentinean provinces [62]. Trace element composition of 25 elements in 74 samples was determined by Inductively Coupled Plasma Mass Spectrometry (ICP-MS). Applying repeated 10-fold cross-validation to optimize each of the classification methods, the results showed that SVM held highest mean accuracy of 76.2% followed by 71% for RF while LDA, KNN, and PLS-DA held the same mean accuracy of 66.7%. RF and SVM also showed 98% and 93% accuracy in determining the geographical origins of grape seeds based on determination of 29 trace elements from Mendoza province in Argentina [63].

A comparison study was performed by Lubinska-Szczygieł et al. [64] on Kaffir (*Citrus hystrix*) and Key (*Citrus aurantifolia*) limes to determine their botanical origin. Dataset samples were produced using two-dimensional GC with time-of-flight MS (GCxGC-TOF-MS). Classification accuracy of four different methods were compared, namely: SVM, classification tree (CT), naïve Bayes (NB), and RF classifications with two-fold cross-validation. The results showed that SVM, NB, and RF statistical models performed with 100% classification accuracy while a CT model performed at 87.5%.

Mineral content of mangoes from uniform genetics (Lippens variety) cultivated in the Gomera Island (Canary Islands) was used for discrimination based on cultivation practices (organic vs. non-organic). Classification of two types of agricultural crops was done by applying LDA and SVM on the samples. The results showed that, while 73.2% classification accuracy is possibly by LDA method, SVM can increase the accuracy up to 93.1%. These findings indicate that, with non-linear boundaries between the classes, ANN is a better classification method than LDA [65].

A low-cost android electronic nose was developed for detection of different types of fruit. Odor patterns were correctly differentiated 100% of the time by kernel extreme learning machine (KELM), producing more accurate results compared to SVM, KNN, LS-SVM, and extreme learning machine (ELM) [66].

Geographical origin classification of Jujube *(Ziziphus jujuba Mill*) fruit was done by evaluating total sugar, acid, phenolic content, and antioxidant activity. Using PCA, LDA, LS-SVM, and BP-ANN classifier models for discrimination of NIR spectra, the results showed that LS-SVM achieved the best results for classification of jujubes [67]. LS-SVM also displayed 100% accuracy in discrimination of Vis/NIR spectroscopy data combined with image processing to detect crack defects of fresh jujube fruit [68]. Munera et al. [69] described a method of using Vis/NIR hyperspectral imaging to determine three stages of persimmon fruit ripeness. Comparison of SVM, LDA, and quadratic discriminant analysis (QDA) showed that at least 94% classification accuracy of the three stages of ripeness was possible by all three methods. The best overall classification of 95.1%, however, was obtained with QDA.

Fresh peaches rapidly deteriorate at ambient summer temperatures. While storage at low temperatures can prolong the shelf life of the fruit, chilling injuries can occur that affect taste quality. A system to detect chilling injury of peaches was developed by pairing hyperspectral reflectance imaging with PLS-DA, ANN, and SVM classifiers [70]. All models obtained high accuracies in a two-class classification set between chilled and non-chilled peaches with ANN and PLS-DA achieving 100% accuracy.

Digital image feature extraction from segmented gray image of grapes illuminated with fluorescent light can be used to discriminate between grapes that were treated with pesticides and untreated grapes. 100% accuracy was achieved by using a linear kernel SVM classifier, showing that image-based processing classification is a good nondestructive method for determining grape pesticide exposure [71].

Most metabolomic studies that deal with classification are focused on two class problems. Multiclass study conducted for classification of 14 raspberry cultivars with varying levels of mold susceptibility, showed poor performance of SVM compared to RF and penalized discriminant analysis (PDA), indicating that SVM algorithms may not be a good method for multiclass classification [72].

Looking to find new methods for real time non-destructive food classification, Zheng et al. [73] explored the use of ELM compared to different chemometric techniques for differentiation between strawberries and other types of fruit. The results showed that SVM achieved 96% accuracy compared to 95.3% for BP-ANN, 95% for ELM, 85% for PLS-DA, and 67% for KNN. These results indicate that SVM had better performance than ELM. Gómez-Meire et al. [74] discussed a comparison among different machine learning techniques, such as SVM, RF, KNN, and NB to find a classification model able to precisely differentiate between existing grape varieties. The authors provided details of the cross-validation method employed (10-fold cross-validation) and of how the training and test sets were defined.

Table 2 summarizes results from the articles describing chemometric applications along with statistical parameters used to compare the different methods and applications for the study of geographical origin, adulteration, ripeness, and quality control parameters of fruits.

#### 2.1.3. Grains

Advancements in technology allow for food crops to be genetically modified (GM) to increase resistance to pests. However, because not all consumers are comfortable eating GM foods, rapid and non-destructive methods to discriminate between GM and non-GM products are needed. Using terahertz spectroscopy (THz) imaging for discrimination of rice transgenic seeds from non-transgenic counterparts, Liu et al. [75] showed that highly accurate prediction models could be created with 96.7% accuracy reported when using RF and 90% when using SVM. An earlier study also showed that with a multispectral imaging (MSI) system, up to 100% classification accuracy could be achieved with LS-SVM and PCA-BPNN models [76].

Sample preparation methods can influence the accuracy of classification. Applying four different preparation methods, namely rice powder pellet with boric acid (RPPBA), rice powder pellet (RPP), rice grain pellet (RGP), and rice grain (RG), Yang et al. [77] correctly classified 20 kinds of rice based on their geographical origin by applying PCA and SVM analysis on data samples generated from laser-induced breakdown spectroscopy (LIBS). Accuracies observed were 92.7% for RPPBA, 95.7% for RPP, 98.8% for RGP, and 99.2% for RG. Data generated by Raman spectroscopy can also be used for classification of rice grain by geographical origin. A classification accuracy of above 90% has been reported by Feng et al. [78], with SVM but requiring more computer resources than KNN.

SVM classifier coupled with data generated from ICP-MS was used to determine concentrations of 19 different trace elements in rice. The study showed that classification between organic and non-organic rice with 98% certainty is possible with this method. Additionally, 96% certainty was accomplished by determining concentrations of only two trace elements, Ca and Cd [79]. Product adulteration is commonly encountered in the food industry but can be detected using this approach. By mixing together pure white rice from Korea and China, adulterated samples were created with various ratios of cross contamination. The results of the study showed that it is possible to discriminate between pure Korean or pure Chinese rice and adulterated samples with as little as 5% contamination. This accuracy was achieved by utilizing RF and SVM on mass spectra from 330 samples of 30 cultivars of Korean and Chinese white rice [80].

Different states of fungal spoilage on brown rice can be monitored by integration of hyperspectral imaging with SOM. This novel method clearly visualized different classes of fungal growth on brown rice [81]. In a study aimed at differentiating between *Lupinus albus* and *Lupinus angustifolius*, SOM proved a reliable method for clustering species and cultivars as well as discovering some new genetic similarities between the two lupin seeds [82].

Taking measurements of 8 experimental indices from 255 durum wheat samples from Sicily, Marini et al. [83] attempted to build a model for reliable classification of durum wheat. Because the classes of the indices slightly overlap, non-linear methods yielded better results compared to the linear methods. MLF-ANN and counter propagation artificial neural network (CP-ANN) resulted in 72.7% and 81.8% correct classification, while linear and quadratic models topped out at a 53%. Collecting data by NIR hyperspectral imaging combined with a quadratic SVM classification tool with a radial basis function (RBF) Gaussian kernel was also shown to be a reliable method for inspecting food safety and quality control [84]. Detection of impurities and contaminants in various types of cereal cultures as well as animal feed can be higher than 95% using this approach. Classification accuracies of 98.9% and 100% were observed using BPNN and LS-SVM methods among six brands of instant noodles using 360 spectra generated by Vis/NIR spectroscopy [85].

Developing a fast and non-destructive method to test for viability of corn seeds in pre- and post-harvest stages is crucial in industrial sorting applications. Using hyperspectral imaging data from a sample size of 600 corn seeds, classification accuracies for corn seed viability using three different chemometric techniques were 97.1% for LDA, 87.9% for PLS-DA, and 100% for SVM [86]. Classifying coated maize kernels on different corn varieties can be done by using NIRS to collect samples. By applying SIMCA, Biomimetic Pattern Recognition (BPR) and SVM chemometric tools Jia et al. [87] showed that at 97.5% classification accuracy, SIMCA outperformed SVM and BPR even though the latter two achieved accuracy above 90%. In a separate study, discriminating between 400 normal and 400 frost-damaged maze kernel samples, prediction accuracies obtained were 94% using SVM, 97.3% using BPR, and 89.5% using Mahalanobis distance (MD) [88]. Parameters such as environmental and cultivation conditions, climate, etc., can deteriorate classification accuracy of models for discrimination of maize seeds when attempting to classify the same type of seed from year to year. To mitigate this problem, Guo et al. [89] suggested periodic updating of the classification algorithm. Using data from hyperspectral images coupled with LS-SVM that achieved 100% classification accuracy on the initial dataset, deterioration of the accuracy of the model over a span of three years was observed to fall in the rage between 53% and 25% for newer samples. While keeping the classification model updated, the study showed that classification accuracy can be maintained at above 87% accuracy, with most samples reaching above 90% accuracy.

In a classification study of five different cultivars of caraway spice, Ghasemi-Varnamkhasti et al. [90] demonstrated that SVM can produce accuracy of 97.9 ± 3.8% and performs better than the LDA model used on the same sample set.

Table 3 summarizes results from the articles describing chemometric applications along with statistical parameters used to compare the different methods and applications for the study of geographical origin, adulteration, discrimination of transgenic and non-transgenic seeds, and quality control parameters of grains.

#### 2.1.4. Protein

Meat processing is one of the largest food processing industries worldwide. Reliable quality control methods are of utmost importance in maintaining high product quality. Generating data samples by Vis/NIR in the range of 400 nm to 1000 nm and NIR in the range of 900 nm to 1700 nm, adulterated minced meat from beef, pork, and chicken can be identified in comparison to unadulterated meat. Using SVM, the overall classification accuracy between the adulterated and unadulterated meats was 96% and 95% for Vis/NIR and NIR [91].

Adulterants added to meat products are a big problem in meat industry. Pork adulteration in veal sausages can be screened by combining NIR with SVM. Methods for laboratory testing, industrial measurement, and on-site analysis were compared by Schmutzler et al. [92]. Meat adulteration was tested in 10% step increments from 100% veal to 50% each veal and pork. Classification of 100% was reported in all tests from 20% to 50% adulteration. At 10% adulteration, a 91.7% classification accuracy was reported in industrial setup when measuring contamination through a plastic package, while an unsatisfactory classification accuracy of 83.3% was recorded using handheld spectrometer in the on-site setup. A method that uses spectral imaging coupled with SVM correctly classified 95.3% of 110 freshly ground samples of pure beef and beef samples adulterated with horse meat. The results also indicated that change of meat color due to storage can significantly affect the performance of this method [93].

Artisan and industrial pork sausages from Brazil were classified in an experiment based on their moisture, protein, fat, nitrite, sodium, and calcium levels. With ANN architecture of six input, five hidden, and two output neurons, a 100% correct classification was accomplished for both classes of pork sausage [94].

Suckling lamb meat can be differentiated according to their rearing system by applying FT-IR spectroscopy to fat samples. Selected features identified either by PCA or SVM were fed into an ANN resulting in 100% correct classification of perirenal fat while PCA extracted features fed to ANN resulted in 9% error in classification of omental fat samples [95].

Combining hyperspectral imaging with SVM techniques has been shown to be a reliable method with 98.2% accuracy for discriminating between organic and conventional raised salmon [96], Applying SVM with data gathered from high resolution ^13^C NMR can be used to predict the farm of origin of farmed salmon [97]. Good performance was also noted when combining Vis/NIR hyperspectral imaging technique with LS-SVM to differentiate between fresh, cold-stored, and frozen-thawed carp fish. The highest CCR of 94.3% was obtained with LS-SVM and probabilistic neural network (PNN) in tandem with first derivative pretreatment. A slightly lower CCR of 91.4% was obtained by a simpler model using LS-SVM and first derivative pre-processing [45]. Raman spectroscopy was used for the classification of caviar in a set of 95 samples containing three different types, in which features such as type and purity were used for classification yielding 93.6% accuracy with multi layered BPNN classification algorithm [98].

Selling meat that has been previously frozen without proper labeling is considered a form of adulteration. A novel method for rapidly differentiating between fresh, previously frozen, and spoiled pork meat utilizes ANN with a three-layer non-linear perceptron applied to data generated from an E-nose based on ultra-fast gas chromatography (UFGC). This method produces classification accuracies of 80%, 85%, and 90% for fresh, frozen then thawed, and spoiled meat, respectively [99]. Li et al. [100] used adaptive boosting orthogonal linear discriminant analysis (AdaBoost-OLDA) machine learning algorithm compared to SVM in an attempt to sense pork meat freshness using a light scattering technique. 100% correct classification was achieved with AdaBoost-OLDA, while SVM algorithm produced classification accuracies of 93.3% for calibration and 96.7% for prediction datasets. AdaBoost also delivered better classification results compared to BP-ANN when determining freshness of pork meat based on total volatile basic nitrogen content [101].

Veterinary drugs such as tetracycline are often found in poultry products due to their use to promote growth and health of industry animals. Residual pharmaceuticals, however, can cause health problems for humans and affect meat quality. Xiao et al. [102] developed a method using synchronous fluorescence spectrometry with SVM to discriminate duck meat with excess tetracycline residues, achieving a 95.7% classification accuracy. Looking to find new methods for real time non-destructive food classification, Zheng et al. [73] explored the use of NIR spectroscopy with different chemometric techniques to differentiate between three classes of fresh minced meats, namely chicken, pork, and turkey. The results showed that ELM achieved 97.8% accuracy compared to 97.7% for PLS-DA, 95.8% for SVM, 95.7% for BP-ANN, and 92.3% for KNN.

Raman micro-spectroscopy combined with SVM was shown to be a reliable and quick method to detect food-borne pathogens. By accessing a Raman spectra database with 19 spices and multiple steps of classification models, an accuracy range from 90.6% to 99.6% in differentiating between Gram-positive and Gram-negative bacteria and bacterial genus can be reached [103].

Table 4 summarizes results from the articles describing chemometric applications along with statistical parameters used to compare the different methods and applications for the study of adulteration, discrimination of organic and conventionally raised fish, and quality control parameters of proteins.

#### 2.1.5. Oils

Edible oils are part of daily diet for humans which makes assessing the quality and authenticity of oils an important issue for the food industry. Combining GC-MS with SVM to analyze the fatty acid composition of 6 different kinds of edible oils resulted in misclassifications of 8.5% for training and 3.0% for test sets [104]. Using SVM and PLS on data generated by FTIR resulted in 100% accuracy for classification of canola, sunflower, corn, and soybean oils [105] as well as distinguishing between pure olive oil and non-olive oil [106,107].

Having an insufficient number of training samples can render machine learning algorithms such as SVM ineffective. However, in some cases, such as swill-cooked dirty oils, the accumulation of adequate data sets is not possible. To overcome that problem, Zhou et al. [108] proposed the use of graph based semi-supervised support vector machine (GS3VM) in an attempt to discriminate between edible and swill-cooked dirty oils. Using data generated by NIR from 100 edible and 99 swill-cooked dirty oils, prediction accuracy by GS3VM method was reported to be 96% for unlabeled and 98% for labeled samples.

Removing variance from sample data as well as optimizing SVM meta-parameters to prevent overfitting can improve the accuracy of SVMs, albeit a time-consuming process. In theory these would be regularization parameter which controls the tradeoff between margin maximization and error minimization and kernel width meta-parameter for the RBF kernel function. To mitigate this, Devos et al. [109] have proposed a method for simultaneous SVM meta-parameter optimization and data preprocessing. The method based on parallel generic algorithm (GENOPT-SVM) was applied to classification of olive oil from the Ligurian region of Italy and olive oils from other Italian regions. The results show classification accuracy improvement from 85.1% to 87.8%, based on an NIR spectral data set and from 74.7% to 82.7% using FTIR spectra. By applying CP-ANN on MS data, prediction accuracy of 84% was obtained for Ligurian olive oil and 76% for non-Ligurian olive oil [110]. However, these accuracies were still lower than the prediction accuracy by NIST’s MS Search program, which is a non-machine learning method, indicating that CP-ANN is highly dependent on features of the training set. An improvement over CP-ANN accuracy was obtained by analyzing GC-MS data with MLP-ANN, resulting in a classification accuracy of 90.1% and a prediction accuracy of 81.1% [111].

Combining LS-SVM with genetic algorithm (GA) and applying it to data generated from THz spectroscopy, Liu et al. [112] obtained 96.3% prediction accuracy in an effort to classifying olive oils from four different regions. Zheng et al. [73] explored the use of NIR with different chemometric techniques to differentiate between authenticated extra virgin olive oils (EVOO) from four different countries of origin: Greece, Italy, Portugal, and Spain, achieving 97.4% accuracy using ELM compared to 95.1% for SVM, 93.1% for PLS-DA, 90.5% for BP-ANN, and 83.3% for KNN. UV-vis spectra can be clustered by SOMs to classify different types of olive oil, which can be used as quality control for discrimination of pure EVOO against refined olive oil and refined olive-pomace oil. Torrecilla et al. [113] obtained a misclassification under 1.3% with SOM based on lag-k autocorrelation coefficients grouping 120 signals into five classes. In a study comparing several different techniques for storage time classification of EVOO, Sanaeifar et al. [114] obtained 100% accuracy with Bayesian network (BN) while ANN with one hidden layer produced accuracy of 97.5% and SVM with a polynomial kernel function achieved accuracy of and 96.3%.

A rapid detection method based on ion mobility spectrometry is available for determining adulteration of sesame oil. Prediction accuracy of 94.2% was reported by applying recursive SVM to discriminate between pure sesame oil and four other types of edible oils [115]. Making use of GC-MS and applying a one-class SVM classifier, the same team reported 100% accuracy in building an authentication model for pure sesame oil [116]. In food quality inspection, a reliable method was developed by Deng et al. [117] to identify different brands of sesame oil in which 100% accuracy was reported when combining SVM with a novel Multiclass Forward Feature Selection algorithm (SVM-MFFS) to analyze data obtained by Vis/NIR.

NIR spectroscopy has shown to be a successful nondestructive method for discrimination of transgenic and non-transgenic soybean oils. By applying SVM Discriminant Analysis, 100% of the samples were correctly classified during the training stage for both types of soybean oils, while 90% and 100% accuracies were reported in validation runs for transgenic and non-transgenic oils [118]. No classification error was reported when using SVM in a study designed for testing the use of FTIR for classification of three varieties of rapeseed oil crop [119]. The authentication of *Rosa damascena* essential oil composition can be done with the use of E-Nose and SVM analysis. A classification accuracy of 99% was reported by Gorji-Chakespari et al. [120] when discriminating between three rose genotypes.

Quality of sandalwood oil from the same species is dependent on geographical origin. SOM techniques applied to NIR spectra showed the ability to correctly differentiate between sandalwood oils from three different geographical regions in India [121].

Table 5 summarizes results from the articles describing chemometric applications along with statistical parameters used to compare the different methods and applications for the study of adulteration, geographical origin, and quality control parameters of oils.

#### 2.1.6. Dairy

Determining freshness of milk and dairy products is of great interest to the industrial and scientific communities. Bougrini et al. [122] assessed the use of multisensor E-Nose and voltammetric E-Tongue by trying to determine the number of storage days for pasteurized milk. A total of 150 samples were generated using five different milk brands, and data taken for pasteurized samples over five storage days (refrigerated at constant temperature of 4 °C) yielded 53.3% classification accuracy for E-nose and 58.7% for E-Tongue. However, perfect classification was obtained when performing mid-level of abstraction data fusion from both E-Nose and E-Tongue, coupling with SVM while using a leave-one-out cross-validation method.

Trace mineral composition can be used for determining authenticity of organic milk. Concentrations of 14 mineral elements in 98 samples of milk from northern Spain were measured by ICP-MS. Making use of an optimized multilayer feed-forward artificial neural network (MLF-ANN), a classification model was developed to discriminate between organic and non-organic milk within a 5% margin of error [123]. However, using trace elements for authentication of milk is highly dependent on geographical origin of the samples, indicating that different prediction models need to be developed for different geographical locations.

Concentrations of illegal adulterants such as water, neutralizers, melamine, etc., can be detected and measured by NIR spectroscopy. From 800 milk samples consisting of 287 raw cow milk samples and 526 adulterated milk samples, Zhang et al. [124] proposed methods for identifying raw and adulterated milk by using pattern recognition methods of improved SVM (I-SVM). This method yields above 94% correct classification at or above a 5% level of adulteration.

Automated microbiological quality evaluation of pasteurized vanilla cream can be performed non-invasively by using FTIR spectroscopy. During pasteurization treatment, microbiological stability of vanilla cream can be compromised, resulting in germination of surviving bacterial spores. One study combined FTIR spectroscopy measurements of samples in addition to sensory evaluation and microbiological determination of aerobic plate count (APC) to form two classes where microbiological data was converted to log (colony-forming units) per gram of cream (log CFU/g): class 1 (accept, APC < 4.5 log CFU/g) and class 2 (reject, APC ≥ 4.5 log CFU/g) [125]. Using SVM classification model with a second-degree polynomial kernel function in tandem with FTIR, spectral fingerprints generated correct classification accuracy of 93.5% for training data sets and 99.2% for the testing data set.

The illegal practice of adding various types of agent, such as detergents, to raw milk to reduce the microbial population poses a serious threat to human health. Detergent powder in raw milk can be detected using an E-Nose based on eight metal oxide semiconductor sensors (MOS) [126]. Adulterated and pure samples of milk were distinguished with 90% accuracy by using SVM with RBF kernel.

Breast milk is an extremely complex sample matrix. For example, composition of breast milk can vary depending on whether a mother is feeding male or female infant. Fatty acids, phospholipids, and tryptophan are found in greater concentrations in mothers feeding female infants while carotenoids and saccharides are more pronounced in milk from mothers having a male infant [127]. One study shows that Raman spectroscopy in tandem with SVM with a second-order polynomial kernel function can distinguish between the two classes of milk with 86% accuracy, 58% sensitivity, and 88% specificity. Contamination of breast milk with polychlorinated biphenyls (PCB), which tend to accumulate in matrixes with high lipid content, poses a health concern to the newborn children [128]. In one study, 193 samples of breast milk from 10 different towns and cities throughout Brazil were analyzed by GC-ECD. A SOM neural network was used to obtain information about variation of PCB contamination in different regions, evaluating proximity to industrial centers, rivers, and the sea as well as whether the mother was breastfeeding for the first time.

Table 6 summarizes results from the articles describing chemometric applications along with statistical parameters used to compare the different methods and applications for the study of adulteration and quality evaluation of dairy food products.

#### 2.1.7. Others

Authenticity of food products is extensively demanded by the consumers and quality control agencies all over the world. Zhu et al. [129] showed how LS-SVM, SVM, BP-ANN, LDA, and KNN were adopted to correctly classify pure and adulterated honey samples. Attempting to perform authentication for the protected designation of origin (PDO) of Galician honey, Latorre et al. [130] developed a method using NIR spectroscopy and various chemometric techniques including MLF-ANN. The data set consisted of 30 honey samples, 15 of which were genuine Galician honey and 15 were trademark commercial and industrially managed honeys from Galician areas. MLF-ANN performed at 100% sensitivity and 93.3% specificity. SIMCA performed at 93.3% sensitivity and 100% specificity, indicating a better rejection of non-genuine honey samples compared to MLF-ANN. Using GCxGC-TOF-MS to analyze profiles of volatile compounds in honey, Stanimirova et al. [131] applied various techniques such as LDA, SIMCA, and SVM for study of honeys based on their geographical origin. The sample set consisted of 374 honeys collected over two years from Corsican and non-Corsican regions. The results showed that SVM had the best performance compared to other methods with 91.5% efficiency, 93.2% sensitivity, and 87.2% specificity. However, the classification model would need to be updated at regular intervals, because variations in samples from year to year would render the model inaccurate in the long run. Applying SVM with RBF kernel to data generated by E-Nose, E-Tongue, NIR, and MIR, Gan et al. [132] concluded that sensor and spectral analysis could be used for classification of botanical origin of honeys as well as detection of honey adulteration. Classification of Brazilian honey by region based on composition of 42 trace elements was investigated by Batista et al. [133], showing that selection of a subset of variables is necessary in order to achieve good results. Comparing MLP-ANN, SVM, and RF classification, the optimal results for classification of honey from the region of São Paulo state compared with honey from other Brazilian regions were recorded when 5 trace elements were used resulting in accuracies of 66.3% for SVM, 79.3% for RF, and 82.8% for MLP-ANN. Moreover, 100% geographical classification of Moroccan and French honeys was achieved using voltammetric E-Tongue coupled with SVM using a leave-one-out cross validation process [134].

Authenticity evaluation of organic Brazilian coffee was performed by determining concentrations of elements using MLP-ANN, SVM, and NB classifiers. MLP-ANN and SVM achieved 96.3% accuracy while NB achieved 98.2% accuracy for discriminating between organic and non-organic coffee [135]. Elemental analysis can also be used for determination of geographical origin of Mexican roasted coffee beans. ICP-OES was used for sample analysis, while LDA and MLP-ANN were used for classification. MLP-ANN achieved a prediction ability of 93% and specificity of 98% while the corresponding metrics for LDA were 81% and 94% [136]. In a separate study designed for classification of arabica coffee by genotypic and geographical origin, Link et al. [137] used RBF-ANN to obtain 100% correct geographic classification and 94.4% genotypic classification. Bona et al. [138] used SVM to produced 100% accuracy for geographical classification of different genotypes of arabica coffee. Looking to find new methods for real time non-destructive food classification, Zheng et al. [73] explored the performance of ELM with standard chemometric techniques in an attempt to differentiate between arabica and robusta coffee species. The results showed that ELM and PLS-DA achieved 100% accuracy compared to 97.5% for SVM, 98.2% for KNN, and 97.5% for BP-ANN.

Machine learning tools have proven to be particularly successful in classification of teas. Several studies achieved 100% accuracy when attempting to classify 3 or more groups of teas using various methods such as BP-MLP-ANN [139], PLS-SOM [140], and probabilistic ANN [141]. Green teas can come in many different assortments attributed to plant varieties and processing methods. 320 images of green tea were captured using multi-spectral imaging and classification accuracies of two LS-SVM classifiers, one with linear kernel and one with RBF kernel, were compared. Achieving 100% classification accuracy, RBF-LS-SVM classifier outperformed LS-SVM which achieved 82.1% accuracy [142]. In an experiment designed to classify Iron Buddha tea by storage period, Xiong et al. [143] showed that when applying LS-SVM and BPNN to data generated by MSI, classification accuracies of 95% and 97.5% are possible for the two methods, respectively. Fuzzy SVM classifier was applied to images taken by a three-charge-coupled device (3-CCD) digital camera by Wang et al. [144] for differentiation between green, oolong, and black tea from China. Because three classes of teas were being classified and SVM classifiers are originally designed to solve two class problems, winner-takes-all method was used to break down three classes into multiple two-class tasks. Overall classification of 97.8% was obtained using this method.

Mineral element content of PDO wine vinegars from three Spanish regions were used to classify vinegars by their geographical origin. ICP-OES was used to establish content of different elements from 25 vinegar samples. Comparison between SVM and LDA classifiers indicated that SVM is a better method that produced 80% classification accuracy while LDA achieved accuracy of 73% [145]. Chinese vinegar samples were used for comparison of RF algorithms against BPNN and SVM. Experiments were performed for three different classification types: different vinegar class grades, vinegar material, and aromatic V-brand which was a small multiclass data set of twelve different Chinese aromatic vinegars. The studies produced statistically complex and unbalanced data sets with classes containing different numbers of samples. For the vinegar-grade class studies, prediction accuracies were 66% for both BPNN and SVM, while RF yielded 98%. BPNN, SVM, and RF achieved 97%, 89.9% and 99% vinegar-material prediction accuracies and 89.0%, 18.9% and 100% for aromatic V-brand. The results showed that RF model outperformed BPNN and SVM for unbalanced, multiclass, and small sample datasets [41]. In other studies, however, SVM classification algorithm proved more reliable. Prediction accuracy by SVM in classification of three types of Spanish PDO vinegar was between 92% and 100% [146]. Above 85% accuracy was reported for identification of mature, aromatic, and rice vinegar when using LS-SVM with RBF kernel [147], while 100% accuracy was reported when classifying sherry vinegar by different aging times [148].

Classification of wine by geographical origin is used for authentication and quality control of products. Trace element concentrations found in wine can be used for this type of investigation. Contents of 17 elements from 64 Spanish white wine samples from four different regions were analyzed by ICP-OES and 100% prediction accuracy was reported using SVM [149]. 272 samples of bottled Slovenian wines were analyzed by ICP-MS and ICP-OES to establish their multielement content. Experimental results showed that CP-ANN model with two layers of neurons performed at 82% accuracy, which the authors considered to be satisfactory due to the small size of Slovenian wine regions [150]. Using Fourier transform ANN, 92.9% accuracy was reported when discriminating against different PDO of wine analyzed by E-Tongue [151]. Gas chromatography (GC) was used for classification of six autochthonous white grape varieties by analyzing volatile aroma compounds from Spanish Galician white wines. Classification capabilities of SVM, RF, MLP-ANN, KNN, and NB were compared on 42 different wine samples. The results showed 100% classification accuracy by RF when all family compounds were used, while MLP-ANN was the best classifier when the amount of available information was reduced [74].

Various machine learning techniques have been investigated for beer quality control. ANN was employed with 100% accuracy in discriminating between good and bad quality of beer based on different features such as alcohol and percentage of carbonation [152]. Similarities and differences in Brazilian Pilsner beers were compared using SOM, and 20 beer brands could be grouped into 6 sets based on the composition of their volatile fractions [153]. Classification of beers based on their geographical origin using SVM showed 99.3% overall prediction ability in distinguishing between beers from Germany, Portugal, and Spain [154].

Chemical information found in metal composition of the orujo distillates was used for development of a system for discrimination between alcoholic distillates with certified brand of origin (CBO) and those without CBO. In comparison between methods, classification accuracy of PNN exceeds that of other techniques such as SVM, resulting in classification accuracies of 98.6 ± 3.1% and 98.0 ± 4.5% for COB and non-COB distillates [155]. Classification of white and rested tequilas was done using SVM on a sample set consisting of 80 bottles with 39 white and 42 rested types classified into 4 sets of white and 4 sets of rested tequilas for a total of 8 groups. Based on these classifications, 14 adulterated samples were correctly identified as fake products [156]. Pérez-Caballero et al. [157], reported classification accuracies of above 94% in differentiating between white, rested, aged, and extra-aged tequilas using RF and SVM. Making use of the ensemble of MLP, SVM and NB, Rodrigues et al. [158] were able to classify Brazilian rum by aging time and wood type used during the aging process. By co-averaging the individual classifiers, accuracies of 100% was achieved for the wood type and 85.7% for aging time.

Classification of raw and processed rhubarb was investigated by Liu et al. [159] by evaluating metabolomic profile of data generated by LC-QTOF-MS. The study showed that PLS-SVM exhibited prediction accuracy of 94.7 ± 7.7%, indicating that this method could be applied for general classification of processed herbal products. A study for classification of three different Indigowoad root samples Radix Isatidis (RI), Rhizoma et Radix Baphicacanthis Cusia (RRBC), and simulated adulterated samples was conducted by Ni et al. [160]. Three pretreatment methods, namely GA-PLS, successive projections algorithm (SPA), and wavelet transform (WT), were compared for selection of the best wavelength variables for NIR spectroscopy. The study also compared methods for each of these pretreatment methods, showing that LS-SVM produced CCRs of 91.0% and 97.2% with GA-PLS and SPA methods, while for a WT pretreatment method Radial Basis Function Artificial Neural Networks (RBF-ANN) and KNN produced CCRs of 97.3% and 98.2%. Fourier transform NIR spectroscopy coupled with SVM was shown to be an excellent technique in classification of cocoa beans. 100% correct classification was reported by Teye et al. [161] in an experiment designed to classify fermented, unfermented, and adulterated cocoa beans. SVM also produced results with 91.8% accuracy in classification of fermented, dried, and unpeeled cocoa beans using Raman spectroscopy [162].

Assuring geographical origin of food is important for both authenticity and quality of products. Many studies have used machine learning classifiers in assessing geographical origin of food products. Ion concentrations and pH values were used for verification of geological origin from 145 samples of bottled mineral water. CP-ANN with supervised learning algorithm was used for prediction of mineral water samples based on four lithological classes, including magmatic rocks, metamorphic rocks, biogenic-chemical sediments, and clastic sediments. Prediction accuracies of 85% and 65% were reported based on recall ability and leave-one-out cross validation algorithms [163]. Fifty-four samples of yerba mate beverage from Argentina, Brazil, Paraguay, and Uruguay were analyzed for trace elements by ICP-OES in an experiment designed to classify the beverage by the country of origin. 100% correct classification for all samples was accomplished using SVM discriminant analysis [164]. 100% classification of Cortex moutan root samples from three different provinces was accomplished by KNN, outperforming LS-SVM and BP-ANN classifiers that produced 94% and 92% prediction accuracies [165]. Tracing the origin of *Marsdenia tenacissima* samples was explored in a study conducted by Li et al. [166]. 27 elements from 128 samples were analyzed with SVM achieving classification accuracies of 98.9% and 100% for training and testing. Similar accuracy of 97% and 93% was reported using PLS-DA and 94% and 90% was reported using SVM classifier for determining the geographical origin of medicinal herbs *A. membranaceus* and *P. albiflora* [167].

Table 7 summarizes results from the articles describing chemometric applications along with statistical parameters used to compare the different methods and applications for the study of adulteration, geographical origin, and quality evaluation of other food groups.

#### 2.1.8. Critical Issues Found with Non-Linear Classification Models in Food Analysis Studies

Classification studies of food products discussed in this review were performed with varying degree of success, using several different linear and non-linear methods. The trend that emerged from the results of these studies is that, in majority of cases, non-linear methods provided better and faster results, compared to linear methods. Among the non-linear methods that were explored, such as different types of ANNs (BP-ANN, FF-ANN, CP-ANN, etc.), Kohonen SOM, and SVM, the SVM classifier has been the most commonly used. The popularity of SVM is inherent in the need to differentiate between two classes, i.e., a binary classification problem, and SVMs were originally developed for that specific purpose. Other advantages of SVMs over other techniques include the ability to select different parameters for kernel functions [168] as well as the capability to handle both linear and non-linear data [169].

Dataset sample size is one of the biggest issues that arises when using non-linear methods for classification problems. A sufficiently large set of data points is required to train machine learning models such as ANNs, SOM, or SVMs. Having a larger number of samples to generate even bigger datasets for training and testing purposes, would be ideal when using non-linear methods for classification. Unfortunately, many studies reviewed here did not use large enough datasets for training of the non-linear algorithms. Rady et al. [170] acknowledged this issue with their own dataset where ANN did not perform as well as LDA and PLS-DA.

In some cases, accuracy of classification models can greatly be impacted by the time frame over which the data was collected. As noted by Hu et al. [58], the ability to differentiate between sound and damaged berries was greatly impacted by the passage of time after the initial mechanical impact. Variation in samples over a prolonged period of time can cause degradation of the classification model [131], thus requiring periodic updates and retraining these models.

Unbalanced datasets are a common issue encountered in many real-world applications as well as instances in which data between classes overlaps, meaning that classes are not linearly separable. To account for this problem, models can utilize pre-sampling methods [171,172] such as oversampling minority classes, undersampling majority classes, random oversampling, dynamic sampling [173], AdaBoost [174], etc.

Sample selection must be considered when designing a classifier. If a dataset contains too much redundant and irrelevant information, then a classifier algorithm may not produce accurate results. Feature subset selection is critical when training machine learning classifiers [175]. In food classification, this technique is especially useful when using trace elements in food to determine geographical origin of the products such as in the study conducted by Batista et al. [133] where results showed that a subset of 5 trace elements yielded better results than using all 42 investigated trace elements.

Very few papers have mentioned or made use of a validation procedure, such as testing the robustness of a model by using external samples or adding some noise to the data. In addition, most papers did not discuss addition of a misclassification penalty when optimizing classification performance [176].

### 2.2. Prediction

Several examples of applications of non-linear models as tools for prediction analysis in food-related problems have been reported in the literature. Unfortunately, most articles did not discuss the assumptions for the use of nonlinear models. In general, the authors compared linear models such as PLS with different types of neural networks (which are inherently non-linear models) and SVMs in terms of predictive capability and statistical measures for goodness-of-fit. The predictive capability can be evaluated by the mean squared error (MSE) or the root mean square error (RMSE). These statistical parameters represent error of prediction and can be derived from the residuals to check the prediction performance of a specific model. When comparing two models, the one with lower MSE is considered to be better. In an ideal scenario MSE would be approaching zero. The RMSE is derived from MSE. It is the standard deviation of the residual. This metrics is a measure of how spread out these residuals are. In an ideal case there would be enough data points to create two independent datasets where one would be used for calibration of the model and the second would be used for validation. It is possible to calculate two kind of RMSE that came from calibration and validation datasets. The first one is the root mean square error for cross-validation (RMSECV) and this validation procedure is known as internal validation. The latter is the root mean square error for validation/prediction (RMSEP) and it is known as external validation. There are other measures of predictive capability that some authors have been using such as relative standard deviation (RSD) of the prediction values, residual predictive deviation (RPD), relative prediction error in percentage (RE%), relative absolute error (RAE), and root relative absolute error. All these metrics represent predictive capability of a model. 

The statistical measures for goodness-of-fit of a model describes how well it fits a dataset. It can be represented by different statistical parameters. In this Section, the goodness-of-fit are described by coefficient of determination (R2), coefficient of correlation (R), and the root mean square error for calibration dataset (RMSEC). The first metric represents the proportion of the variance in the dependent variable that is explained from independent variable. The second one measures the strength and the direction of a linear relationship between two variables. The last one is RMSEC for calibration dataset. In the below mentioned papers, all these statistical parameters are primarily used for evaluating the accuracy of measurement in food analysis. Generally, a model with a good prediction ability should have large R or R2, and small RMSEC, RMSECV, RMSEP, RSD, RPD, RE%, RAE, and root relative absolute error. Detailed mathematical descriptions for all these metrics can be found in the book ‘Multivariate Calibration’ by Martens et al. [177].

#### 2.2.1. Vegetables

Content of bioactive compounds in food can be used as a method for sorting and grading of crops. The use of multispectral imaging combined with chemometric methods for determining content of lycopene and phenolic compounds in intact tomatoes was investigated by Liu et al. [178]. Their findings indicated that the BPNN prediction model is superior to LS-SVM, with R2 of 0.938 and RPD of 4.6 for lycopene while (R2) of 0.97 and RPD of 9.3 were observed for total phenolics content.

Niu et al. [179] describe a method to determine the quantity of glucose and fructose in lotus root powder. The optimal model was obtained by LS-SVM, which gave the best result when compared with other methods like PLSR and BP-ANN. Rady et al. [50] have developed a prediction model for evaluation of sugar content in potatoes using PLSR. In a separate study, the BP-ANN considerably improved the prediction performance of color change and moisture distribution in carrot slices during hot air dehydration when compared with PLS and LS-SVM [180]. All these works described the method of cross-validation employed, with the leave-one-out being the most common. The description of training, test, and calibration sets was detailed as well.

On the other hand, several works lacked the information or had very few details about the cross-validation methods applied and how the training, test, and calibration sets were built. Some studies applied different regression models [181,182,183,184,185,186], and although the researchers present excellent results, the capability of the models to predict new samples is unclear due to lack of deep discussion about using the data with respect to cross-validation methods, validation process, and training, test, and calibration split methods employed.

Table 8 summarizes results from the articles describing chemometric applications with statistical parameters that were discussed for prediction of vegetables.

#### 2.2.2. Fruits

Several authors have used different non-linear methods to study fruits. Wei et al. [170] and Li et al. [187] determined sugar content, pH, and firmness of pears by comparison of different linear and non-linear regression analysis. The comparison was realized in terms of coefficient of correlation, coefficient of determination, and RMSEP. In both works, LS-SVM was superior to the PLS method in predicting sugar, pH, and firmness in pears. Das et al. [188] tested three different kernel models to construct SVM models for calculation of convective heat transfer coefficient to investigate pear drying performance. The accuracy of the models was checked by RMSE, relative absolute error, and root relative absolute error. The normalized polynomial kernel performed better than other SVM kernel models for estimating the convective heat transfer coefficient values.

Several studies used comparisons of linear and non-linear models to quantify quality properties of different fruits. For instance, Conesa et al. [189], Guo et al. [67,190], Cao et al. [191], and Malegori et al. [192] used spectroscopy for evaluation of soluble solids and other properties of fruits. All these studies indicated that non-linear methods produced best quantitative prediction results. Therefore, spectroscopic techniques in conjunction with non-linear models can be a very useful and promising alternative to the traditional laboratory techniques for monitoring properties of fruits. Sanaeifar et al. [193] were able to determine total soluble solids and other quality properties of banana in different shelf-life stages by application of a low-cost electronic nose with measurement technique. The dataset was analyzed with linear and non-linear methods to predict these properties.

Firmness is another quality attribute of fruits studied by researchers, where non-linear methods and linear models have been used. Firmness is related to the maturity of the fruit and can be an indicator of product’s shelf life, and as such is a key factor for consumers when purchasing fruit in deciding whether the product is fresh and of high quality. Zhu et al. [194] applied linear and non-linear methods calibration to establish firmness of peaches using PLS and SVM approaches. In this study, the linear method with variable selection by competitive adaptive reweighted sampling (CARS) algorithm showed better results than SVM model. Another work where PLS showed better results than SVM in determining firmness was conducted by Xue et al. [195] to analyze Chinese pear-leaved crabapple.

Other comparisons of linear and non-linear regression aimed at checking the quality attributes of fruit, include mechanical properties [196], astringency [197], browning levels [198], total anthocyanin content [199], antioxidant activity [199], and food additives [200]. Taking into account the lessons drawn from the above mentioned papers on fruit analysis, all of these studies in followed the good practice suggested by Marini [201], to start by determining whether linear models give good results and then switching to non-linear methods to compare the results. Linear and non-linear methods were used to find the best fit without considering the nature of the data. Some authors such as Niu et al. [179] and Mariani et al. [202] have discussed the intrinsic non-linearity in the data as well. Niu et al. evaluated glucose and fructose in lotus root powder based on FT-NIR spectroscopy and concluded that LS-SVM model is better than linear models because non-linearity in the spectral data or in the chemical nature of glucose and fructose in lotus root powder was apparent. On the other hand, Xue et al. found that PLS model is better than SVM for determining firmness in Chinese pear-leaved crabapple for the first day and the fourth day of the shelf life. However, with the extension of shelf life both linear and non-linear models did not work anymore. This indicates that glucose and fructose are changing during fruit ripening process and the dataset is showing this process. Mariani determined soluble solid content in fruit by NIR and concluded that LS-SVM was able to find the non-linear relationships between soluble solid content and the NIR data.

Table 9 summarizes results from the articles describing chemometric applications with statistical parameters that were discussed in regression of fruits.

#### 2.2.3. Grains

Grains are important sources of many nutrients, including fiber, B vitamins (thiamin, riboflavin, niacin, and folate), carbohydrates, protein, and minerals (iron, magnesium and selenium). Peng et al. [203] compared linear and non-linear methods, in terms of RMSEP, to build models with NIR spectra of corn to determine moisture, oil, protein, and starch contents. In this work, the authors used the linear PLS method and a non-linear method called ELM. They proposed an extension of ELM algorithm by linear and nonlinear functions to describe the regression relationship between concentrations of these substance and NIR spectra. The results showed that non-linear methods outperform the linear method. Other authors used non-linear methods to study rice [204,205,206,207]. Abbasi-Tarighat et al. [204] applied spectrophotometric method to the simultaneous determination of Mn^2+^ and Fe^3+^ in different kinds of food including rice, with data analysis by radial basis function networks (RBFNs) and FFNNs. The results showed that the proposed method is simple, provides a wider linear range, and lower RSD%.

Zhang et al. [208] used THz spectroscopy and compared the results obtained by SVM and PLS models to simultaneously determine amino acid mixtures in cereal using different preprocessing. In this work, SVM models can be considered as the best method for data preprocessing because results obtained showed lower RMSECV and RMSEP and higher R2 for majority of amino acids mixtures. Das et al. [188] compared different linear and non-linear models to monitor changes in sucrose, reducing sugar, and total sugar content due to water-deficit stress in rice by spectroscopic analysis using ANN, multivariate adaptive regression splines (MARS), random forest regression (RFR), SVM, multiple linear regression (MLR), and PLSR. The best results were obtained with non-linear models for all three of these properties with respect to R2, RMSEC, and RMSEP. The relationship of sugars with spectral data was better described by non-linear methods, which is consistent with other previous results in the literature [209,210]. Fu et al. [211] used LS-SVM on data obtained by fourier transform near infrared (FT-NIR) spectroscopy for the analysis of a toxic additive, maleic acid, in cassava starch. The findings from this study indicate that these methods allow for rapid evaluation and can be used for other applications such as untargeted analysis.

Table 10 summarizes results from the articles describing chemometric applications with statistical parameters that were discussed in the prediction of grains.

#### 2.2.4. Protein

Widely varying methods have been proposed and employed for the evaluation of freshness or incipient spoilage in food that are high in protein. Li et al. [212] used BPNN and SVM to build prediction models of yolk index with a dataset obtained by electronic nose. The SVM model with reduction of dataset by independent component analysis (ICA) showed better results than BPNN. Many authors have studied fish with non-linear methods and instrumental analysis. Papadapoulos et al. [213] have used BPNN for the determination of chlorinated compounds in fish. Xu et al. [214] have used PLSR and epsilon-support vector regression to create a technique for rapid and accurate determination of fish caloric density. In both studies, relatively small datasets used for training and testing could represent a generalization problem of the techniques. Vis/NIR hyperspectral imaging technique can also be used for determining freshness of grass carp fish fillets by measurement of total volatile basic nitrogen (TVB-N) content. LS-SVM model was shown to give better performance than PLS regression with R2 of 0.92 and 0.91 and RMSEP of 2.35% and 2.75% for the two methods, respectively. The method produced even better results when using SPA to select nine optimal wavelengths achieving R2 of 0.91 and RMSEP of 2.78% [215].

Papadopoulou et al. [216] have used SVM to perform a sensory and microbiological quality assessment of beef fillets. Clear information was presented with regards to data selection and cross-validation technique, with some discussion about overfitting. Similarly, Prevolnik et al. [217] have used ANN to predict pork drip loss from pH and color measurements of near infrared spectra, describing clearly how the set training and testing sets were selected as well as the method applied for cross-validation.

Table 11 summarizes results from the articles describing chemometric applications with statistical parameters that were discussed in the prediction of protein.

#### 2.2.5. Oils

Yang et al. [218] analyzed the oil content of rapeseed by applying ANN method on data generated by NIR. The study showed that multilayer feed-forward neural networks with 8 nodes (MLFN-8) are the most suitable and reasonable mathematical model to use, with a RMSEP of 0.59. Cabrera and Prieto [219] used artificial neural networks for the prediction of the antioxidant activity of essential oils. Results showed that ANN are reliable, fast, and cheap tools for predicting antioxidant activity of essential oils and can also be used to model biochemical properties of complex natural products and predict the quality of food ingredients. Sanaeifar et al. [114] used several non-linear models (ANN, SVM, BN) and the MLR linear model to investigate quality of olive oil during storage. Results showed that SVM with RBF kernel had the best performance-based correlation coefficient for prediction of peroxide value, UV absorbance at 232 nm, and chlorophyll. Dong et al. [220] evaluated adulteration of extra virgin olive oil using Raman spectroscopy data using linear and non-linear models, with Bayesian framework LS-SVM (Bay-LS-SVM) providing higher accuracy, i.e., good predictive capability and appropriate goodness of fit.

Zhang et al. [221] studied measurement of aspartic acid by NIR in oilseed rape leaves under herbicide stress using linear and non-linear methods and concluded that the best model was generated using SVM. Riahi et al. [222] compared MLR, PLS, polynomial PLS (poly-PLS), and SVM to construct a quantitative relation between the retention index of some essential oil components and their calculated molecular descriptors. The results obtained from the data indicated that SVM was best-fitted model.

Table 12 summarizes results from the articles describing chemometric applications with statistical parameters in prediction of oils.

#### 2.2.6. Dairy

Non-linear methods have been applied to predict many different properties of interest in dairy products. Bassbasi et al. [223] determined solid non-fat content in raw milk by Attenuated Total Reflectance-Fourier Transform Infrared spectroscopy (ATR-FTIR) and methods including PLS and SVM. However, the authors did not discuss the assumptions used for the SVM model, but compared the R2, RE%, RMSEC, RMSECV, and RMSEP between PLS and SVM models. The non-linear model showed better results than PLS with RE% between 0.39% and 0.29%, depending on the spectral range. Wei et al. [224] used SVM and PLS models to evaluate the ability of voltammetric electronic tongue (VE-tongue) to predict the rheological (viscosity), acidic (pH), and time characteristics in different periods (fermentation, post-ripeness, and storage stages) of set yogurt in terms of R2 and RSD of the validation values. Both models efficiently predicted the pH, viscosity, and storage time during the storage process, but PLS performed better than SVM. Other examples of non-linear regression compared with PLS as applied to dairy products are provided by Rocha et al. [225], Altieri et al. [226], and Wu et al. [227], but the authors from all three papers only compared the models by R2, RMSEP, and RE% and did not discuss dataset non-linearity. On the other hand, Balabin and Smirnov [228] discussed the non-linearity of data in dairy products and have compared many linear and non-linear multivariate calibration models for melamine detection in liquid milk, infant formula, milk powder based on vibrational spectroscopy, NIR, and MIR. The authors concluded that the relationship between the MIR/NIR spectrum of milk products and melamine content is nonlinear because the non-linear models presented RMSEP values three times lower than linear models.

Table 13 summarizes results from the articles describing chemometric applications with statistical parameters that were discussed in prediction of dairy food.

#### 2.2.7. Others

Foods classified as “others” are defined in the Section 1.4 of this paper as a set consisting of food such as beverages, water, spices, etc., that did not fit in the previous six groups. Several papers describe the application of different methods of linear and non-linear regression models such as PCA, PLS, SVM, and ANN. Tan et al. [229] have demonstrated the use of an ensemble strategy that employs a combination of SOM and PLS techniques for NIR spectral calibration. The results of this technique displayed good accuracy when using data from complex beverage samples. However, no discussion or comparison was presented as to whether the accuracy of the model could be improved using non-linear methods.

Ni et al. [181] have developed a procedure for determination of aminocarb and carbaryl in vegetable and water samples by applying classical least squares (CLS), PLS, PCR, BP-ANN, RBF-ANN, and PC-RBF-ANN. All these methods were applied for the prediction of the carbamate pesticides in vegetable and water samples. The results showed that PLS and PC-RBF-ANN calibration models gave the lowest prediction errors. Wu et al. [230] compared PLS against the non-linear methods BP-ANN and LS-SVM to evaluate the feasibility of using NIR spectroscopy for determining three antioxidant activity indices of bamboo leaf extract. Neither paper provided information about the assumptions chosen to define the training and testing sets.

Ouyang et al. [231] proposed a novel cross-perception multi-sensor data fusion approach to predict human panel test results. The non-linear methods SVM and BP-ANN achieved R2 > 0.8 for E-eye, E-nose and E-tongue methods while MLR achieved R2 > 0.8 only for E-tongue method. Other works [232,233] have detailed the use of linear and non-linear methods in order to build regression models with excellent results. Nevertheless, these works lack a discussion about cross-validation techniques for avoiding overfitting of the designed models. Absence of this information does not diminish the importance of the results achieved; however, they prevent a more in-depth analysis of how those models would behave when used on external data.

On the other hand, Liu et al. [234] have presented the results of using LS-SVM to determine acetic, tartaric, and lactic acids in plum vinegar based on Vis/NIR. In this work, the authors chose a leave-one-out cross-validation method to avoid overfitting. The work of many others [235,236,237,238,239] has also presented enough information about training and testing sets, cross-validation methods, and overfitting considerations, providing good examples for the use of these models.

Table 14 summarizes results from the articles describing chemometric applications with statistical parameters that were discussed in the prediction of other foods.

#### 2.2.8. Critical Issues Found with Non-Linear Prediction Models to Study Food Analysis

Different nonlinear prediction models have been employed to study food analysis. Techniques such as SVM, BPNN, RBF-ANN, and others were listed during this study. In general, the papers analyzed in this review compare nonlinear and linear models through statistical parameters such as R2, RMSEC, RMSECV, RMSEP, RSD, and RE%. These comparison criteria are the most common. Another way to do this comparison is by applying statistical significance testing before making conclusions whether a nonlinear model is better than a linear model. Significance tests show the level of statistical confidence which indicates whether a difference truly exists between linear and non-linear methods. Examples of some tests which can be used to compare the two models are F-test [240], Aikake information criteria (AIC) [241,242], and Bayes information criteria (BIC) [243]. Unfortunately, only few studies in this review have demonstrated the use of a significance test to determine whether one model is superior over another. Furthermore, some authors performed these comparisons based on statistical significance testing using small datasets split into calibration (training) and validation (testing) datasets. This strategy to evaluate the performance of linear and non-linear methods and the assumption of independence, which is usually required for statistical tests, is not valid. In these cases, bootstrap methods [244] and/or cross validation procedures [245] are advisable to build test models before comparing the linear and non-linear models.

Special attention should be given to the comparison between the models made through the R2, as this metric alone is an inadequate measure of how well linear and non-linear models fit the data. Nevertheless, R2 is frequently used within the food science literature for the analysis and interpretation of data fitting. In some cases, a low R2 value may be determined for a good model, or a high R2 value for a model that does not fit the data. When using the R2 to compare models, supplementation with other statistical methods such as checking residual plots for random behavior, drawing a graph with all observations, checking if the dataset has outliers, and considering the subject area knowledge is required to conclude that one model is better than another [246].

Another critical issue found was the use of insufficient data (training, testing, and validation sets) when building regression models, which is directly related to a model’s ability of generalization. As majority of the papers studied for this review did not present any discussion about how a cross-validation technique was employed, and therefore some models tend to present an overfitting behavior. In order to avoid the problem of overfitting, some form of validation [247] must be employed, such as testing the model with a set of data completely independent from the training set, or using an internal cross-validation approach. Validation processes for the use of non-linear and linear models is essential in food science because application of these models often needs to be approved by federal government agencies, such as MAPA, Anvisa, USFDA, and USDA. Thus, the models should be validated with regards to precision, accuracy, absence of bias, standard error of prediction, prediction interval, signal-to-noise ratio, limit of detection, limit of quantification, sensitivity, and selectivity. Most of the papers used in this review did not discuss such validation.

The final issue is uncertainty estimation in food science. According to De Bièvre [248], a result without a reliability (uncertainty) statement cannot be trusted. In general, majority of papers in this review have not described their procedure on how to deal with uncertainty estimation of the models used. Many papers in the literature [249,250,251] tackle the issue of uncertainty estimation in linear and non-linear methods, such as bootstrap and jackknifing analysis [251], which could be applied in the area of food science.

## 3. What Changes have Happened between 2008 and 2018 in the Area of Food Analysis That Facilitate the Application of Non-Linear Methods?

Several changes have occurred during the last decade among the scientific community in the area of food analysis that facilitate the application of non-linear methods.

- Many instruments used for generating analytical data are equipped with software that performs chemometric analysis by non-linear methods. While these applications may not be totally optimized, these software bundles assist in dissemination of the non-linear methods to analysts, i.e., the people who generate the data.
- Increasingly, studies are being developed with a multidisciplinary team of chemists, biologists, engineers, and data scientists in the food area. The data scientist, after understanding the problem that needs be studied and the questions which are required to be answered by the analysts, can adjust the linear or non-linear models more adequately for extracting useful information from the data.
- Increasingly, user-friendly software and free code libraries written by data scientists, mathematicians, and statisticians are available on the internet with algorithms that create non-linear models. This availability of information enables researchers who are not familiar with computer programming to use non-linear models and allows a greater number of researchers to apply non-linear models to their data without the need for a deep knowledge of the algorithms. Consequently, this increases the dissemination of non-linear methods in various scientific communities in food analysis.

## 4. Summary from Classification and Prediction

Through the examination of summary tables from the articles discussed in this review, it is possible to note that accuracy of ANN models is highly dependent on sample-set size. This can be seen from the pattern that emerges from a number of studies such as the ones conducted by Rady and Guyer [50], Palacios-Morillo et al. [55], Liu et al. [76], Liu and He [85], and Marini et al. [83]. The increase in the number of samples, from 255 by Marini et al. [83] to 400 samples Liu et al. [76], as well as Zheng et al. [73] using around 10 times as many samples than Guo et al. [67] to build their models resulted in an increased accuracy of ANN models. On the other hand, SVM models showed less variability in performance accuracy based on the sample set size. Above 90% classification accuracy rates were reported in experiments conducted by Feng et al. [78], Barbosa et al. [79], and Jia et al. [87], where dataset sizes were below 100 samples. Identical accuracy was reported by Yang et al. [77], Wakholi et al. [86], Jia et al. [88], Guo et al. [89], and Ghasemi-Varnamkhasti et al. [90], who built SVM models with much larger sample sets that ranged from 600 to 3208.

When comparing ANN classifiers with SVM, Zheng et al. [73] had worse performance when compared to SVM models. However, when the number of samples for the construction of classifier model was increased, ANN was able to deliver a better performance than SVM [41,114,143].

With respect to linear models, such as AdaBoost–OLDA, LDA, and PLS-DA, it can be observed that for smaller sample sets in the range of 60–90 samples, linear models perform just as well as non-linear models. In experiments conducted by Zheng et al. [73], Li et al. [100], and Huang, et al. [101], reported accuracies for non-linear classifiers were above 90% and, in a study for determining the geographical origin of medicinal herbs *A. membranaceus* and *P. albiflora* [167], PLS-DA performed even better than SVM.

From the results obtained about models used for prediction analysis in food-related problems showed in Table 8, Table 9, Table 10, Table 11, Table 12, Table 13 and Table 14, it is possible to conclude that non-linear models performed better than linear ones as it was previously discussed [229,231,232,233,234,235,236,237,238] in Section 2.2.7. However, linear models can achieve high accuracy when used with a small number of samples. Examples are highlighted in the papers that discuss PLS [195,197,214] and MLR [230] models.

Accuracy of SVM models is not as highly dependent on sample-set size. However, it is worth highlighting that the SVM model performed better than the PLS, even with a small number of samples [208,223,224].

## 5. Conclusions and Future Perspectives

Non-linear methods are versatile and flexible tools for modelling complex relationships among complicated datasets obtained from various types of instrumental analysis. These methods have widely been applied in food analysis for classification and regression studies. Many examples have been studied over ten years (2008–2018), and their performance compared with traditional methods showed that non-linear methods are able to achieve results of high quality that, in some cases, are not obtainable with the traditional methods. Many researchers are increasingly using various non-linear methods for the construction of models which are more adequate and accurate in solving problems of regression and classification. This indicates that researchers understand that non-linear phenomena occur in nature, and the best way to investigate them is through the application of models that capture this information more adequately.

This review has been able to show ideas about application of non-linear methods that have become relatively commonplace in food analysis. While this field is still developing, with the growth of computers in power and speed, new methods and variations are more widely available. A large variety of websites are offering free downloads and software packages to encourage use of non-linear methods. The reader is encouraged to ponder the advantages and disadvantages of these methods in practical applications and to choose the most suitable methods for analysis of their experimental data in order to extract important patterns, trends, and to understand “what the data say”.

## Figures and Tables

**Table 1:** Literature related to the use of chemometrics in classification of vegetables.

Sample/Application Description	Chemometric Method(s)	Number of Samples (Total)	Statistical Parameters	Ref Num
Classification of Mushroom origin	IC-OVO-LS-SVM	1800	Accuracy = 93.2% Sensitivity = 93.1% Specificity = 99.7%	[42]
Classification of Tomato Genotypes	LS-SVM, DA, SIMCA	283	Accuracy: 100% for all methods	[46]
Classification of tomato juice freshness	SVM, BPNN, Cluster-then Label	150	Accuracy: SVM = 94.2% BPNN = 97.0% Cluster-then-Label = 98.7%	[48]
Quality of processed potato chips	LS-SVM	80	RMSECV: Fat Content = 0.201 Moisture Content = 0.065 Acid value = 0.068 Peroxide = 0.369	[49]
Classification of potatoes based on sugar levels	ANN, LDA, PLS-DA	990	Accuracy: ANN = 78.0% LDA = 79% PSLDA = 81%	[50]
Identification of foodborne pathogens contamination in packaged fresh vegetable	SOM	120	Accuracy = 97.5%	[51]
Classification model for geographical traceability of mushrooms	SVM	65	Accuracy = 90.91%	[52]
Discrimination of Boletus mushrooms by geographical origin	SVM	332	Accuracy: training = 99.1% testing = 100%	[54]
Classification of paprika by geographical origin	MLP-ANN	2016	Sensitivity = 99% Specificity = 99%	[55]
Classification of cassava roots	ANN, KNN, SVM	no clear information	not shown but referenced as supplementary information	[56]

**Table 2:** Literature related to the use of chemometrics in classification of fruit.

Sample/Application Description	Chemometric Method(s)	Number of Samples (Total)	Statistical Parameters	Ref Num
Orange juice adulteration	BPNN, SVM	108	Accuracy: BPNN = 100% SVM = 100%	[41]
Classification of bayberries based on presence of bruises	PC-SVM, SVM	112	Fractal parameters accuracy: SVM-F = 100% PC-SVM = 100% RGB parameters accuracy: SVM = 85.29%	[57]
Classification of blueberry damage with time evolution	MP-ANN	737	Sound blueberry accuracy: Reflectance method = 94.7% Transmittance method = 94.7% Interactance method = 85.5% Damaged blueberry accuracy: Reflectance method = 77.8% Transmittance method = 100% Interactance method = 100%	[58]
Discrimination of strawberry juice	RF, SVM	20 samples × 5 groups	Accuracy: RF: e-nose data = 96% e-tongue data = 100% fusion of E-nose and E-tongue data = 100% SVM: e-nose data = 84% e-tongue data = 100% fusion of E-nose and E-tongue data = 88%	[59]
Detection of infection in date fruit	SIMCA, PLS-DA, PCA-ANN	408	Accuracy: SIMCA = 82% PLS-DA = 93% PCA_ANN = 86%	[60]
Geographical origin of chayote fruit	LDA, KNN, PLS-DA, SVM	92	Accuracy: LDA = 89.1% KNN = 84.7% PLS-DA = 82% SVM = 87%	[61]
Geographical origin of lemon juice	LDA, KNN, PLS-DA, RF, SVM	74	Mean accuracy: LDA = 66.7% KNN = 66.7% PLS-DA = 66.7% RF = 71% SVM = 76.2%	[62]
Determining geographical origins of grape seeds	RF, SVM	408	Accuracy: RF = 98% SVM = 93%	[63]
Botanical origin of limes	CT, NB, RF, SVM	no clear indication of number of samples	Accuracy: CT = 87.5% NB = 100% RF = 100% SVM = 100%	[64]
Discrimination between organic and non-organic mangoes	LDA, SVM	130	Accuracy: LDA = 73.2% SVM = 93.1%	[65]
Classification of fruit by type	KNN, LS-SVM, SVM, ELM, KELM	400	Accuracy: KNN = 93.75 LS-SVM = 97.5% SVM = 97.5% ELM = 97.5% KELM = 100%	[66]
Geographical origin classification of Jujube	LS-SVM, BP-ANN	97	Accuracy: LS-SVM = 93.8% BP-ANN = 81.2%	[67]
Detection of crack defect in jujube fruit	LS-SVM	176	Accuracy: LS-SVM = 100%	[68]
Classification of persimmon ripeness	LDA, QDA, SVM	90	Overall accuracy ± standard deviation: LDA = 90.2 ± 7.6 QDA = 95.1 ± 4.1 SVM = 90.3 ± 9.7	[69]
Classification of chilled and non-chilled peaches	PLS-DA, ANN, SVM	330	Accuracy: PLS-DA = 100% ANN = 100% SVM = 96.55%	[70]
Discrimination between grapes treated with pesticides and untreated grapes	SVM	72	Accuracy: SVM = 100%	[71]
Classification of 14 different cultivars of a single raspberry species	RF, PDA, PLS, SVM		Classification error: PTR-MS data: RF = 0.187 PDA = 0.282 PLS = 0.299 SVM = 0.257 GC-MS data: RF = 0.213 PDA = 0.202 PLS = 0.266 SVM = 0.223	[72]
Differentiation between strawberries and other types of fruit	KNN, PLS-DA, ELM, BP-ANN, SVM	983	Accuracy: KNN = 67% PLS-DA = 85% ELM = 95% BP-ANN = 95.3% SVM = 96%	[73]
Differentiation between existing grape varieties	SVM, RF, KNN, MLP, NB	42	mean kappa coefficient: SVM F-value = 10.347, df = 7, p-value = 6.56E - 9 RF F-value = 2.607, df = 7, p-value = 0.019 KNN F-value = 1.854, df = 7, p-value = 0.09 MLP F-value = 3.614, df = 7, p-value = 0.0022 NB F-value = 2.104, df = 7, p-value = 0.054	[74]

**Table 3:** Literature related to the use of chemometrics in classification of grains.

Sample/Application Description	Chemometric Method(s)	Number of Samples (Total)	Statistical Parameters	Ref Num
Discrimination of rice transgenic and non-transgenic seeds	RF, SVM	200	Accuracy: RF = 96.7% SVM = 90%	[75]
Discrimination of rice transgenic and non-transgenic seeds	PLSDA, LS-SVM, PCA-BPNN	400	Accuracy: PLDA = 98% LS-SVM = 100% PCA-BPNN = 100%	[76]
Rice classification by Geographical origin	PCA-SVM	2000	Accuracy: 99.2%	[77]
Classification of rice grain by geographical origin	KNN, SVM	42	Accuracy: Above 90%	[78]
Discrimination between organic and non-organic rice	SVM	50	Accuracy = 96% Specificity = 100% Sensitivity = 88%	[79]
Adulteration detection in rice	RF, SVM	330	Predictive performance at 5% adulteration: RF Accuracy = 0.8 Sensitivity = 0.8 Specificity = 0.8 Positive prediction value = 0.8 Negative prediction value = 0.8 SVM Accuracy = 0.9 Sensitivity = 1 Specificity = 0.8 Positive prediction value = 0.83 Negative prediction value = 1	[80]
Classification of fungal growth on brown rice	SOM	210	No clear metric provided	[81]
Discrimination between two species of lupin	SOM	No clear number provided	No clear metric provided	[82]
Classification of durum wheat	MLF-ANN, CP-ANN	255	Predictive ability: MLF-ANN = 72.7% CP-ANN = 81.8%	[83]
Classification of impurities from different origins in cereals	SVM	112	various classification rates in range 95% to 98.28%	[84]
Detection of impurities and contaminants in various types of cereal cultures	BPNN, SVM	360	Accuracy: BPNN = 98.9% LS-SVM = 100%	[85]
Classifying viability of corn seeds in pre- and post-harvest stages	LDA, PLS-DA, SVM	600	Accuracy: LDA = 97.1 PLS-DA = 87.9% SVM = 100%	[86]
Classification of coated maize kernels	SIMCA, BPR, SVM	40	Accuracy: SIMCA = 97.5% BPR = 91.25% SVM = 90%	[87]
Detection of damage and viability assessment of maize seed	MD, BPR, SVM	800	Accuracy: MD = 89.5% BPR = 97.3% SVM = 97.3%	[88]
Yearly model updating for classification of maize seeds	LS-SVM	800	Accuracy: Initial = 100% Over time with model updating = 87% to 90%	[89]
Classification of caraway cultivars	LDA, SVM	3208	Accuracy: LDA = 96.74 ± 4.36% SVM = 97.92 ± 3.82%	[90]

**Table 4:** Literature related to the use of chemometrics in classification of protein.

Sample/Application Description	Chemometric Method(s)	Number of Samples (Total)	Statistical Parameters	Ref Num
Discrimination of fresh from cold-stored and frozen-thawed fish	LS-SVM, PNN, CCR	120	Accuracy: PNN = 94.29% LS-SVM = 91.43%	[45]
Classification of minced meats	ELM, PLS-DA, SVM, BP-ANN, KNN	60	Accuracy: ELM = 97.8% PLS-DA = 97.7% SVM = 95.8% ANN = 95.7% KNN = 92.3%	[73]
Identification of adulterated minced meat	SVM	1697	Accuracy: SVM with Vis-NIR data = 96% SVM with NIR data = 95%	[91]
Meat Adulteration	SVM	84	Accuracy: 20% and above adulteration on all setups SVM = 100% 10% adulteration Industrial setup SVM = 91.7% On site setup SVM = 83.3%	[92]
Meat Adulteration	SVM	110	Accuracy: SVM = 95.3%	[93]
Discrimination between artisan and industrial pork sausages	ANN	90	Accuracy: ANN = 100%	[94]
Classification of suckling lamb meat	ANN	106	Accuracy on perirenal fat sample: ANN = 100% Misclassification of omental sample: ANN = 9–13%	[95]
Discrimination between organic and conventionally raised salmon	SVM	160	Accuracy: SVM = 98.2%	[96]
Classification of farmed salmon by farm origin	SVM	59	Accuracy: SVM with GC data = 96.61–100% SVM with NMR data = 96.6–100%	[97]
Classification of caviar purity	BPNN	95	Accuracy: BPNN = 93.6%	[98]
Differentiating between fresh, previously frozen, and spoiled pork	ANN	1008	Accuracy: ANN fresh sample = 80% ANN frozen then thawed = 85% ANN spoiled meat = 90%	[99]
Determining freshness of the meat	AdaBoost–OLDA, LDA, SVM	90	Accuracy: LDA = 90% SVM = 96.67% AdaBoost–OLDA = 100%	[100]
Determining freshness of the meat	AdaBoost–OLDA, BP-ANN	77	AdaBoost–OLDA: Rp = 0.8325 RMSEP = 6.9439 BP-ANN: Rp = 0.7946 RMSEP = 6.4343	[101]
Classification of Tetracycline Residue in Duck Meat	SVM	70	Accuracy: SVM = 95.7%	[102]
Identification of meat-associated pathogens		4622	Accuracy: SVM across hierarchical cluster analysis ranges from 90.6% to 99.6%	[103]

**Table 5:** Literature related to the use of chemometrics in classification of oils.

Sample/Application Description	Chemometric Method(s)	Number of Samples (Total)	Statistical Parameters	Ref Num
Classification of olive oil by geographical location	ELM, SVM, PLS-DA, BP-ANN, KNN	60	Accuracy: ELM = 97.4% SVM = 95.1% PLS-DA = 93.1% BP-ANN = 90.5% KNN = 83.3%	[73]
Classification of edible vegetable oils	SVM	66	Misclassification rate: Training set = 8.5% Test set = 3%	[104]
Classification of edible oils	SVM-DA, PLS-DA	103	Accuracy: SVM-DA = 100% PLS-DA = 100%	[105]
Classification of blended olive oil	SVM	146	Accuracy: Olive oil sample = 100% Vegetable oil sample = 92%	[106]
Differentiating olive oil from other edible vegetable oils	SVM	127	Accuracy: SVM = 98%	[107]
Discrimination between edible oil and swill-cooked dirty	GS3VM	199	Accuracy: Labeled samples = 96% Unlabeled samples = 98%	[108]
Classification of Italian olive oil	GENOPT-SVM	910	Accuracy: NIR dataset = 87.8% FTIR dataset = 82.7%	[109]
Classification of Italian olive oil	CP-ANN	220	Accuracy: Ligurian sample = 84% Non-Ligurian sample = 76%	[110]
Classification of Ligurian and non-Ligurian olive oil	MLP-ANN	914	Recognition rate = 90.1% Prediction rate = 81.1%	[111]
Discrimination of geographical origin of extra virgin olive oils	LS-SVM, BPNN	320	Accuracy: Calibration set LS-SVM = 100% BPNN = 100% Prediction set LS-SVM = 96.25% BPNN = 86.25%	[112]
Detection of adulterations in extra virgin olive	SOM	120	Misclassification: Less than 1.3%	[113]
Storage time classification of olive oil	BN, ANN, SVM	393	Accuracy: BN = 100% ANN = 97.5% SVM = 96.3%	[114]
Detection of adulteration of sesame oil	R-SVM	210	Accuracy at above 10% adulteration: R-SVM = 94.2%	[115]
Detection of adulteration of sesame oil	SVM	80	Accuracy: SVM = 100%	[116]
Identification of different brands of sesame oil	SVM-MFFS	120	Accuracy: SVM-MFFS = 100%	[117]
Discrimination of transgenic and non-transgenic soybean oils	SVM-DA	80	Accuracy: Transgenic sample = 90% Non transgenic sample = 100%	[118]
Classification of three varieties of rapeseed oil crop	SVM	120	Accuracy: SVM = 100%	[119]
Authentication of Rosa damascena essential oil composition	SVM	210	Accuracy: SVM = 99%	[120]
Classification of sandalwood oils from three different geographical regions	SOM	49	Accuracy: SOM = 100%	[121]

**Table 6:** Literature related to the use of chemometrics in classification of dairy food.

Sample/Application Description	Chemometric Method(s)	Number of Samples (Total)	Statistical Parameters	Ref Num
Determining the number of storage days for pasteurized milk	SVM	150	Accuracy: Colaimo sample = 96.67% Saiss sample = 100%	[122]
Determining authenticity of organic milk	MLF-ANN	98	Error: MLF-ANN = around 5%	[123]
Determination of illegal adulterants in milk	SVM	800	Accuracy at or above 5% adulteration: SVM = 94%	[124]
Quality evaluation of pasteurized vanilla cream	SVM	97	Accuracy: SVM training data = 93.5% SVM testing data = 99.2%	[125]
Detecting detergent powder in raw milk	SVM	16 samples × 6 group	Accuracy: SVM = 90%	[126]
Distinguish between the two classes breast of milk	SVM	190	Accuracy: SVM = 86% Specificity: SVM = 88% Sensitivity: 55%	[127]
Identification of breast milk by environmental conditions of the living place	SOM	193	Successful visual separation of samples	[128]

**Table 7:** Literature related to the use of chemometrics in classification of other food groups.

Sample/Application Description	Chemometric Method(s)	Number of Samples (Total)	Statistical Parameters	Ref Num
Recognition of Chinese vinegar	BPNN, SVM, RF	432	Accuracy: BPNN = 87.74% SVM = 66.51% RF = 99.8%	[41]
Differentiation between arabica and robusta coffee species	ELM, PLS-DA, SVM, KNN, BP-ANN	56	Accuracy:ELM = 100% PLS-DA = 100% SVM = 97.5% KNN = 98.2% BP-ANN = 97.5%	[73]
Assuring the authenticity of northwest Spain white wine	RF, MLP-ANN	42	Performance: RF 100% accuracy with full feature sets MLP-ANN best when using reduced feature set	[74]
Authentication of honey by geographical origin	LS-SVM, SVM, BP-ANN	135	BP-ANN Specificity = 90% Sensitivity = 90.5% Accuracy = 90.2% SVMSpecificity = 85% Sensitivity = 85.7% Accuracy = 85.4% LS-SVM Specificity = 100% Sensitivity = 91.3% Accuracy = 95.1%	[129]
Authentication of Galician honey	MLF-ANN, SIMCA	30	MLF-ANN Sensitivity = 100% Specificity = 93.3% SIMCA: Sensitivity = 93.3% Specificity = 100%	[130]
Tracing the geographical origin of honeys	LDA, SIMCA, SVM	374	LDA: Sensitivity = 86.4% Specificity = 82.1% SIMCA: Sensitivity = 93.2% Specificity = 45.2% SVM: Sensitivity = 93.2% Specificity = 87.2%	[131]
Classification of botanical origin and adulteration detection of raw honey	SVM	259	No clear metric	[132]
Classification of Brazilian honey by region	MLP-ANN, SVM, RF	57 samples and 42 chemical elements	Accuracy: MLP-ANN = 82.8% SVM = 66.3% RF = 79.3%	[133]
Geographical classification of Moroccan and French honeys	SVM	47	Accuracy: SVM = 100%	[134]
Controlling the authenticity of organic coffee	SVM, MLP-ANN, NB	54	Accuracy: SVM = 96.3% MLP-ANN = 96.3% NB = 98.2%	[135]
Characterization of Mexican coffee	LDA, MLP-ANN	51	MLP-ANN Prediction ability = 93% Specificity = 98% LDA Prediction ability = 81% Specificity = 94%	[136]
Classification of arabica coffee by genotypic and geographical origin	RBF-ANN	90	Accuracy: RBF-ANN Geographic = 100% Genotypic = 94.4%	[137]
Geographical classification of different genotypes of arabica coffee	SVM	74	Accuracy: SVM = 100%	[138]
Differentiation of tea varieties	BP-MLP-ANN	90	BP-MLP-ANN: Sensitivity = 100% Specificity = 100%	[139]
Classification of Chinese tea varieties	PLS-SOM	no clear number	Accuracy: PLS-SOM = 100%	[140]
Classification of teas	ANN	30	Accuracy: ANN = 100%	[141]
Classification of green teas	RBF-LS-SVM, LS-SVM	320	Accuracy: RBF-LS-SVM = 100% SVM = 82.1%	[142]
Classification of Iron Buddha tea by storage period	LS-SVM, BPNN	180	Accuracy: LS-SVM = 95% BPNN = 97.5%	[143]
differentiation between green, oolong, and black tea	SVM	300	Accuracy: SVM = 97.8%	[144]
Characterization of Andalusian wine vinegars	LDA, SVM	28	Accuracy: LDA = 73% SVM = 80%	[145]
Authentication of Spanish PDO wine vinegars	SVM	79	Accuracy: SVM = 92.9 - 100%	[146]
Identification of mature, aromatic, and rice vinegar	LS-SVM	95	Accuracy: LS-SVM = 85% -100%	[147]
Classification of sherry vinegar by different aging times	LS-SVM	57	Accuracy: LS-SVM = 100%	[148]
Classification of Spanish white wines by geographical location	SVM	64	Accuracy: SVM = 100%	[149]
Classification of Slovenian wines by geographical regions	CP-ANN	272	Accuracy: CP-ANN = 82%	[150]
Discrimination of different wine Denominación de Origen	ANN	71	Accuracy: ANN = 92.9%	[151]
Classification of beer quality	ANN	70	Accuracy: ANN = 100%	[152]
Classification of beer brands based on the composition of their volatile fractions	SOM	60	SOM successful grouping of 20 brands into 6 sets	[153]
Classification of beers based on their geographical origin using	SVM	68	Prediction ability: SVM = 99.3%	[154]
Classification of orujo distillate alcoholic samples according to their certified brand of origin	PNN, SVM	115	Recognition ability: CBO distillate PNN = 98.6% SVM = 100% Non CBO distillate PNN = 98.0% SVM = 100% Prediction ability: CBO distillate PNN = 87.7% SVM = 77.9% Non CBO distillate PNN = 86.1% SVM = 71.7%	[155]
Classification of white and rested tequilas	SVM	80	Accuracy: SVM = 100%	[156]
Classification to differentiate white, rested, aged and extra-aged tequila	SVM, SVM-RFE	170	Accuracy: SVM on White tequila = 100% SVM on Rested tequila = 89% SVM on Aged tequila = 94% SVM-RFE = 94%	[157]
Classification of Brazilian rum by aging time and wood type used during the aging process	MLP, SVM, NB	150	Wood type recognition accuracy: MLP = 99.04% SVM = 99.04% NB = 97.14% Ensemble = 100% Recognition of aging time accuracy: MLP = 69.52% SVM = 78.38% NB = 68.57% Ensemble = 85.71%	[158]
Classification of raw and processed rhubarb	PLS-SVM	73	Accuracy: PLS-SVM = 94.7%± 7.7%,	[159]
Classification of three different Indigowoad root samples	RBF-ANN, LS-SVM, KNN	75	Best average correct classification ratios: RBF-ANN = 97.3% LS-SVM = 97.2% KNN = 98.2%	[160]
Classification of cocoa beans	SVM	132	Accuracy: SVM = 100%	[161]
Classification of fermented, unfermented, and adulterated cocoa beans	SVM	500	Accuracy: 91.8%	[162]
verification of the geographical origin of commercially sold mineral water	CP-ANN	145	Correct prediction rate: CP-ANN recall ability = 85% CP-ANN leave-one-out cross validation = 65%	[163]
Classification of yerba mate beverage by country of origin	SVM	54	Accuracy: SVM = 100%	[164]
Classification of Cortex mouton root samples from three different provinces	KNN, LS-SVM, BP-ANN	77	Accuracy: KNN = 100% LS-SVM = 94% BP-ANN = 92%	[165]
Determining the geographical origin of the medicinal plant Marsdenia tenacissima	SVM	128	Accuracy: SVM training = 98.9% SVM testing = 100%	[166]
Determining the geographical origin of medicinal herbs	PLS-DA, SVM	85	Accuracy: A. membranaceus sample PLS-DA = 97% SVM = 94% P. albiflora sample PLS-DA = 93% SVM = 90%	[167]

**Table 8:** Literature related to the use of chemometrics in prediction of properties of vegetables.

Sample/Application Description	Chemometric Method(s)	Number of Samples (Total)	Statistical Parameters	Ref Num
Predicting the content of bioactive compounds in intact tomato fruit	PLS, LS-SVM, BP-ANN*	162	RMSEC = 0.112 RMSEP = 0.308 $R_{P}^{2}$ = 0.965 $R_{C}^{2}$ = 0.998 RPD = 9.335	[178]
Quantitative analysis of glucose and fructose in lotus root powder	PLSR, BP-ANN, LS-SVM*	Glucose = 76 Fructose = 77	Glucose RMSEC = 0.107% RMSEP = 0.115% rc = 0.9827 rp = 0.9765 RPD = 4.599 Fructose RMSEC = 0.543% RMSEP = 0.812% rc = 0.9243 rp = 0.8286 RPD = 1.785	[179]
Determination of color change and moisture distribution in carrot slices	PLS, LS-SVM, BP-ANN*	700	RMSEP = 1.482% $R_{P}^{2}$ = 0.991 RPD = 11.378	[180]
Determination of aminocarb and carbaryl in vegetable and water samples	LS, PLS*, PCR, BP-ANN, RBF-ANN, PC-RBF-ANN	20	relative prediction errors (%RPET): PLS = 5.0 PC-RBF-ANN = 4.8	[181]
Modeling the drying kinetics of green bell pepper in a heat pump	BP-ANN		RMSE = 5.5E-05 $R_{P}^{2}$ = 0.99828	[184]
Chemometric methods for rapid detection of sucrose adulteration in tomato paste	PLS, LS-SVM*, BP-ANN	50	RMSEP = 0.445% $R_{P}^{2}$ = 0.966 RPD = 5.014	[185]
Rapid detection of Escherichia coli contamination in packaged fresh spinach	PCA, BP-ANN*	150	MSE = 0.038 $R_{P}^{2}$ = 0.97	[186]

* indicates the best model from which statistical parameters are displayed in this table.

**Table 9:** Literature related to the use of chemometrics in prediction of properties of fruit.

Sample/Application Description	Chemometric Method(s)	Number of Samples (Total)	Statistical Parameters	Ref Num
A comparative study for the quantitative determination of soluble solids content	PLS, LS-SVM*	480	rc = 0.9286 rp = 0.9164 RMSEC = 0.2113 RMSEP = 0.2506	[187]
Investigation of Pear Drying Performance by Different Methods	SVM	378	RMSEP = 0.3351	[188]
An Electrochemical Impedance Spectroscopy System for Monitoring Pineapple Waste Saccharification	PLS, BP-ANN*	200	RMSEP = 1.206 $R_{P}^{2}$ = 0.970	[189]
Evaluation of chemical components and properties of the jujube fruit	PCA, LDA, LS-SVM*, BP-ANN	97	rc = 0.910 RMSEC = 0.10 rp = 0.904 RMSEP = 0.26	[67]
Determination of soluble solids content of ‘Fuji’ apple	ICA-SVM	160	rp = 0.9455 RMSEP(%) = 0.3691	[190]
Soluble solids content and pH prediction and varieties discrimination of grapes	Genetic Algorithm (GA)	439	Prediction rate = 96.58% $R_{P}^{2}$ = 0.9781	[191]
Evaluation of acerola fruit quality,	PLS, SVM*	117	RMSEP = 0.16 $R_{P}^{2}$ = 0.72 RMSEC = 0.11 $R_{C}^{2}$ = 0.78	[192]
Prediction of banana quality properties	PLS, MLR, SVR*	#	RMSEP = 0.1523 $R_{P}^{2}$ = 0.7607 RMSEC = 0.0722 $R_{C}^{2}$ = 0.9518	[193]
Study on the quantitative measurement of firmness distribution maps at the pixel level inside peach pulp	PLSR	200	RMSEP = 5.176 RMSEC = 4.465	[194]
Study of Malus Asiatica Nakai’s firmness during different shelf lives	PLS*, PCR, LS-SVM	240	RMSEP = 0.5856 rp = 0.7494	[195]
Prediction of mechanical properties of blueberry	SNV	429	rp = 0.91 rc = 0.91 RMSEP = 0.0325 RMSEC = 0.0482	[196]
Prediction of the level of astringency in persimmon	PLSR*, SVM, LS-SVM	130	$R_{P}^{2}$ = 0.904 RMSEP = 0.705	[197]
Rapid detection of browning levels of lychee pericarp	PLSR, BP-ANN, RBF-SV*	360	RMSEP (%) = 0.83% $R_{P}^{2}$ = 0.948 RMSEC (%) = 0.80% $R_{C}^{2}$ = 0.946	[198]
chemometrics for predicting total anthocyanin content and antioxidant activity of mulberry fruit	PLSR, LS-SVM*	180	$R_{P}^{2}$ = 0.995 RPD = 14.255 RMSEC = 0.049 RMSECV = 0.159	[199]
The prediction of food additives in the fruit juice	SVM, RF*, ELM*, PLSR	120	RF: RMSEP = 0.3377 $R_{P}^{2}$ = 0.9105 RMSEC = 0.2727 $R_{C}^{2}$ = 0.9246 ELM: RMSEP = 0.1358 $R_{P}^{2}$ = 0.9141 RMSEC = 0.1776 $R_{C}^{2}$ = 0.9783	[200]

* indicates the best model from which statistical parameters are displayed in this table.

**Table 10:** Literature related to the use of chemometrics in prediction of properties of grains.

Sample/Application Description	Chemometric Method(s)	Number of Samples (Total)	Statistical Parameters	Ref Num
Combination of activation functions in extreme learning machines for multivariate calibration	CELM	215	RMSEP = 0.2780	[203]
Method to the simultaneous determination of Mn2+and Fe3+infoods, vegetable and water sample	RB-ANN*, BP-ANN	39	$R_{P}^{2}$ = 0.9997 RMSEP = 0.74	[204]
Prediction of 2-acetyl-1-pyrroline content in grains of Thai Jasmine rice	PLS	#	RMSECV = 0.091 $Q^{2}$ = 0.8470	[205]
Predict components of starch and protein in rice	PLS, LS-SVM*	320 (starch)320 (protein)	Starch: rp = 0.946 RMSEP = 0.198 Protein: rp = 0.974 RMSEP = 0.071	[206]
Quantitative monitoring of sucrose, reducing sugar and total sugar dynamics for phenotyping of water-deficit stress tolerance in rice	BP-ANN, MLR, PLS, SVMR* and others	144	$R_{P}^{2}$ = 0.99 RMSEP = 2.45	[207]
Simultaneous determination of amino acid mixtures in cereal	PLS, SVM*	32	RMSECV(%) = 0.7303 $R_{CV}^{2}$ = 0.8618 RMSEP(%) = 0.9018 $R_{P}^{2}$ = 0.9732	[208]
Optimizing the tuning parameters of least squares support vector machines regression for NIR spectra	LS-SVM	420	RMSEP = 9.99 $R_{P}^{2}$ = 0.91	[209]
Screening and quantification of maleic acid in cassava starch	LS-SVM	165	RMSECV(%) = 0.208 RMSEP(%) = 0.192	[211]

* indicates the best model from which statistical parameters are displayed in this table.

**Table 11:** Literature related to the use of chemometrics in prediction of properties of protein.

Sample/Application Description	Chemometric Method(s)	Number of Samples (Total)	Statistical Parameters	Ref Num
Prediction of egg storage time and yolk index	BP-ANN, ICA-SVM*	140	RMSEC = 0.0112 RMSEP = 0.0255 $R_{P}^{2}$ = 0.9707 $R_{C}^{2}$ = 0.9730	[212]
Determination of chlorinated compounds in fish	BP-ANN	27	RMSEC = 0.0240 RMSEP = 0.0358	[213]
Determination of fish caloric density	PLSR*, epsilon-SVR	151	nRMSEC = 7.501% nRMSEP = 6.871% nRMSECV = 7.821% rc = 0.874 rp = 0.908 rcv = 0.862	[214]
Determination of TVB-N content for freshness evaluation of grass carp	LS-SVM*, PLSR	120	RMSEC = 1.987% RMSEP = 2.346% RMSECV = 2.2355% $R_{P}^{2}$ = 0.916 $R_{C}^{2}$ = 0.934 $R_{CV}^{2}$ = 0.921	[215]
Sensory and microbiological quality assessment of beef fillets	SVM	177	Prediction rate = 89% $R_{P}^{2}$ = 0.86 $R_{C}^{2}$ = 0.96	[216]
An attempt to predict pork drip loss from pH and colour measurements or near infrared spectra using artificial neural networks	BP-ANN, CP-ANN*	312	RMSEC = 2.3% RMSEP = 2.6% $R_{P}^{2}$ = 0.28 $R_{C}^{2}$ = 0.53	[217]

* indicates the best model from which statistical parameters are displayed in this table.

**Table 12:** Literature related to the use of chemometrics in prediction of properties of oils.

Sample/Application Description	Chemometric Method(s)	Number of Samples (Total)	Statistical Parameters	Ref Num
Analysis of the Oil Content	BP-ANN	29	RMSEP = 0.59	[218]
Prediction of the antioxidant activity of essential oils	BP-ANN	30	Medim relative error = 3.16%	[219]
Quantitative analysis of adulteration of extra virgin olive oil	LS-SVM	39	RMSEP = 0.0509 RMSEC = 0.0201 $R_{P}^{2}$ = 0.9976 $R_{C}^{2}$ = 0.9996	[220]
Measurement of aspartic acid in oilseed rape leaves under herbicide stress	SPA-LS-SVM	248	RMSEP = 0.0339 RMSEC = 0.0428 $R_{P}^{2}$ = 0.9962 $R_{C}^{2}$ = 0.9936	[221]
Investigation of different linear and nonlinear chemometric methods for modeling of retention index of essential oil components	SVM	100	$SE_{C}=1.96\%$ $SE_{P}=4.95\%$ $R_{P}^{2}$ = 0.962 $R_{C}^{2}$ = 0.987 $R_{CV}^{2}$ = 0.963	[222]

**Table 13:** Literature related to the use of chemometrics in prediction of properties of dairy food.

Sample/Application Description	Chemometric Method(s)	Number of Samples (Total)	Statistical Parameters	Ref Num
FTIR-ATR determination of solid non fat (SNF) in raw milk	PLS, SVM*	56	RMSEP = 0.29 RMSEC = 0.21 $R_{P}^{2}$ = 0.998	[223]
Monitoring the fermentation, post-ripeness and storage processes ofset yogurt	PLSR, SVM*	210	$RSD_{C}$ = 0.86% $RSD_{P}$ = 1.13% $R_{P}^{2}$ = 0.9738 $R_{C}^{2}$ = 0.9895	[224]
Quantification of whey in fluid milk	BP-ANN	30	RMSEP = 2.6639 RMSEC = 0.21 $R_{P}^{2}$ = 0.9999 $R_{C}^{2}$ = 0.9935	[225]
On-line measure of donkey’s milk properties by near infrared spectrometry	PLS	178	RMSEP = 0.40 FSPERR = 3.3%	[226]
Study on infrared spectroscopy technique for fast measurement of protein content in milk powder	LS-SVM	410	RMSEP = 0.4115 RMSEC = 0.21 $R_{P}^{2}$ = 0.981 $R_{C}^{2}$ = 0.9935	[227]
Melamine detection by mid- and near-infrared (MIR/NIR) spectroscopy	PLS, Poly-PLS*, BP-ANN, LS-SVM	69	RMSEP = 1.3	[228]

* indicates the best model from which statistical parameters are displayed in this table.

**Table 14:** Literature related to the use of chemometrics in prediction of properties of other food types.

Sample/Application Description	Chemometric Method(s)	Number of Samples (Total)	Statistical Parameters	Ref Num
An ensemble method based on a self-organizing map for near-infrared spectral calibration of complex beverage samples	SOMEPLS*, PLS, KSPLS	218	RMSEP = 3.50	[229]
Determination of antioxidant activity of bamboo leaf extract	PLS, MLR*, BP-ANN, LS-SVM	66	RMSEP = 4.621 rp = 0.966 RMSEC = 3.252	[230]
Instrumental intelligent test of food sensory quality	MLR, SVM, BP-ANN*	75	rc = 0.9392 RMSEC = 1.88 rp = 0.9060 RMSEP = 2.27	[231]
Quantitative determination of aflatoxin B1 concentration in acetonitrile	PLS, PCR, SVM, PCA-SVM*	160	Prediction accuracy = 93.75%	[232]
Application of successive projections algorithm for variable selection to determine organic acids of plum vinegar	SPA-LS-SVM*, MLR, PLS	225	RMSEC = 0.2851 RMSEP = 0.3581	[234]
determination of total antioxidant capacity and total phenolic content of Chinese rice wine	PLS, SVM*	222	RMSEP = 17.94 $R_{P}^{2}$ = 0.9529 RMSEC = 16.59 $R_{C}^{2}$ = 0.9572	[235]
Determination of effective wavelengths for discrimination of fruit vinegars	PLS-DA, LS-SVM*	240	RMSEP = 0.083 $R_{P}^{2}$ = 0.995 RMSEC = 0.028 $R_{C}^{2}$ = 0.999	[236]
Investigating the discrimination potential of linear and nonlinear spectral multivariate calibrations for analysis of phenolic compounds	PLS, PRM, BP-ANN*	61	RMSEC = 0.34 $R_{C}^{2}$ = 0.9945 RMSEP = 0.34 $R_{P}^{2}$ = 0.9811	[237]
Optimization of NIR calibration models for multiple processes in the sugar industry	SVM*, PLS	1797	RMSEP = 0.084	[238]
Quality grade discrimination of Chinese strong aroma type liquors	Combined PLS-SVM	108	Prediction accuracy = 92.6% RMSEC = 0.084 $R_{C}^{2}$ = 0.990 RMSEP = 0.180 $R_{P}^{2}$ = 0.953	[239]

* indicates the best model from which statistical parameters are displayed in this table.

## References

[1] (2019). Food Safety and Quality. http://www.fao.org/food-safety/background/en/.

[2] Odeyemi O.A. (2019). Food Safety Knowledge, Attitudes and Practices among Consumers in Developing Countries: An International Survey. Food Res. Int..

[3] (2019). Ministry of Agriculture, Livestock and Food Supply. https://www.gov.br/agricultura/pt-br/internacional/english.

[4] (2019). About the U.S. Department of Agriculture. https://www.usda.gov/our-agency/about-usda.

[5] (2019). New Era of Smarter Food Safety. https://www.fda.gov/food/new-era-smarter-food-safety.

[6] Efenberger-Szmechtyk M., Nowak A., Kregiel D. (2018). Implementation of Chemometrics in Quality Evaluation of Food and Beverages. Crit. Rev. Food Sci. Nutr..

[7] Skoog D.A., Holler F.J., Crouch S.R. (2007). Principles of Instrumental Analysis.

[8] Despagne F., Massart D.L. (1998). Neural Networks in Multivariate Calibration. Analyst.

[9] Trevor Hastie R.T., Friedman J. (2009). The Elements of Statistical Learning: Data Mining, Inference, and Prediction.

[10] Von Davier A.A. (2017). Computational Psychometrics in Support of Collaborative Educational Assessments. J. Educ. Meas..

[11] Marsman M., Borsboom D., Kruis J., Epskamp S., van Bork R., Waldorp L.J., Maris G. (2018). An Introduction to Network Psychometrics: Relating Ising Network Models to Item Response Theory Models. Multivar. Behav. Res..

[12] Varian H.R. (2014). Big Data: New Tricks for Econometrics. J. Econ. Perspect..

[13] (2014). Laser Spectroscopy for Sensing Fundamentals, Techniques and Applications.

[14] Benitez J.M., Castro J.L., Requena I. (1997). Are artificial neural networks black boxes?. IEEE Trans. Neural Netw..

[15] Jure Z., Johann G. (1999). Neural Networks in Chemistry and Drug Design: An Introduction.

[16] Agatonovic-Kustrin S., Beresford R. (2000). Basic concepts of artificial neural network (ANN) modeling and its application in pharmaceutical research. J. Pharm. Biomed. Anal..

[17] Svozil D., Kvasnicka V., Pospichal J. (1997). Introduction to multi-layer feed-forward neural networks. Chemom. Intell. Lab. Syst..

[18] Montana D.J., Davis L. (1989). Training Feedforward Neural Networks Using Genetic Algorithms. InIJCAI.

[19] Kohonen T. (1995). Self-Organizing Maps.

[20] Tian J., Azarian M.H., Pecht M. (2014). Anomaly Detection Using Self-Organizing Maps-Based K-Nearest Neighbor Algorithm.

[21] Vesanto J., Himberg J., Alhoniemi E., Parhankagas J. (2000). SOM toolbox for Matlab 5.

[22] Vapnik V. (1995). The Nature of Statistical Learning Theory.

[23] Syarif I., Prugel-Bennett A., Wills G. (2016). SVM Parameter Optimization using Grid Search and Genetic Algorithm to Improve Classification Performance. Telkomnika.

[24] Joachims T. (2002). Learning to Classify Text Using Support Vector Machines: Methods, Theory and Algorithms.

[25] Kramer W. (1985). The Power of the Durbin-Watson Test for Regressions without an Intercept. J. Econ..

[26] Halunga A.G., Orme C.D., Yamagata T. (2017). A heteroskedasticity robust Breusch-Pagan test for Contemporaneous correlation in dynamic panel data models. J. Econ..

[27] Thursby J.G. (1982). Misspecification, Heteroscedasticity, and the Chow and Goldfeld-Quandt Tests. Rev. Econ. Stat..

[28] Shapiro S.S., Wilk M.B. (1965). An Analysis of Variance Test for Normality (Complete Samples). Biometrika.

[29] Solari M.E. (1967). Chakravarti, Im—Handbook of Methods of Applied Statistics. Nature.

[30] Ghosh S. (1987). Note on a Common Error in Regression Diagnostics Using Residual Plots. Am. Stat..

[31] Larsen W.A., Mccleary S.J. (1972). Use of Partial Residual Plots in Regression-Analysis. Technometrics.

[32] Huber-Carol C., Balakrishnan N., Nikulin M., Mesbah M. (2012). Goodness-of-Fit Tests and Model Validity.

[33] (2020). How to Identify the Distribution of Your Data. https://statisticsbyjim.com/hypothesis-testing/identify-distribution-data.

[34] (2020). Probability Plot. https://www.itl.nist.gov/div898/handbook/eda/section3/probplot.htm.

[35] (2019). Know Your Food Groups. https://www.nia.nih.gov/health/know-your-food-groups.

[36] Wold S., Sjostrom M., Eriksson L. (2001). PLS-regression: A basic tool of chemometrics. Chemom. Intell. Lab. Syst..

[37] Fernandes J.A., Irigoien X., Goikoetxea N., Lozano J.A., Inza I., Pérez A., Bode A. (2010). Fish recruitment prediction, using robust supervised classification methods. Ecol. Model..

[38] Thomas C., Balakrishnan N. (2008). Improvement in minority attack detection with skewness in network traffic. Data Mining, Intrusion Detection, Information Assurance, and Data Networks Security.

[39] Zhu X., Davidson I. (2007). Knowledge Discovery and Data Mining: Challenges and Realities.

[40] Khoshgoftaar T.M., Yuan X., Allen E.B. (2000). Balancing Misclassification Rates in Classification-Tree Models of Software Quality. Empir. Softw. Eng..

[41] Liu M., Wang M., Wang J., Li D. (2013). Comparison of random forest, support vector machine and back propagation neural network for electronic tongue data classification: Application to the recognition of orange beverage and Chinese vinegar. Sens. Actuators B Chem..

[42] Fu H., Yin Q., Xu L., Wang W., Chen F., Yang T. (2017). A comprehensive quality evaluation method by FT-NIR spectroscopy and chemometric: Fine classification and untargeted authentication against multiple frauds for Chinese Ganoderma lucidum. Spectrochim. Acta Mol. Biomol. Spectrosc..

[43] Zhang G.P. (2009). Neural Networks for Data Mining. Data Mining and Knowledge Discovery Handbook.

[44] Forina M., Casale M., Oliveri P., Lanteri S. (2009). CAIMAN brothers: A family of powerful classification and class modeling techniques. Chemom. Intell. Lab. Syst..

[45] Cheng J.-H., Sun D.W., Pu H.B., Chen X., Liu Y., Zhang H., Li J.L. (2015). Integration of classifiers analysis and hyperspectral imaging for rapid discrimination of fresh from cold-stored and frozen-thawed fish fillets. J. Food Eng..

[46] Xie L., Ying Y., Ying T. (2009). Classification of tomatoes with different genotypes by visible and short-wave near-infrared spectroscopy with least-squares support vector machines and other chemometrics. J. Food Eng..

[47] Gil-Sánchez L., Soto J., Martínez-Máñez R., Garcia-Breijo E., Ibáñez J., Llobet E. (2011). A novel humid electronic nose combined with an electronic tongue for assessing deterioration of wine. Sens. Actuators A Phys..

[48] Hong X., Wang J., Qi G. (2015). E-nose combined with chemometrics to trace tomato-juice quality. J. Food Eng..

[49] Ni Y., Mei M., Kokot S. (2011). Analysis of complex, processed substances with the use of NIR spectroscopy and chemometrics: Classification and prediction of properties—The potato crisps example. Chemom. Intell. Lab. Syst..

[50] Rady A.M., Guyer D.E. (2015). Evaluation of sugar content in potatoes using NIR reflectance and wavelength selection techniques. Postharvest Biol. Technol..

[51] Siripatrawan U. (2008). Self-Organizing algorithm for classification of packaged fresh vegetable potentially contaminated with foodborne pathogens. Sens. Actuators B Chem..

[52] Li Y., Zhang J., Li T., Liu H., Li J., Wang Y. (2017). Geographical traceability of wild Boletus edulis based on data fusion of FT-MIR and ICP-AES coupled with data mining methods (SVM). Spectrochim. Acta A Mol. Biomol. Spectrosc..

[53] Silvestri M., Bertacchini L., Durante C., Marchetti A., Salvatore E., Cocchi M. (2013). Application of data fusion techniques to direct geographical traceability indicators. Anal. Chim. Acta.

[54] Yao S., Li T., Li J., Liu H., Wang Y. (2018). Geographic identification of Boletus mushrooms by data fusion of FT-IR and UV spectroscopies combined with multivariate statistical analysis. Spectrochim. Acta A Mol. Biomol. Spectrosc..

[55] Palacios-Morillo A., Jurado J.M., Alcázar Á., de Pablos F. (2014). Geographical characterization of Spanish PDO paprika by multivariate analysis of multielemental content. Talanta.

[56] Uarrota V.G., Moresco R., Coelho B., da Costa Nunes E., Peruch L.A.M., de Oliveira Neubert E., Maraschin M. (2014). Metabolomics combined with chemometric tools (PCA, HCA, PLS-DA and SVM) for screening cassava (Manihot esculenta Crantz) roots during postharvest physiological deterioration. Food Chem..

[57] Lu H., Zheng H., Hu Y., Lou H., Kong X. (2011). Bruise detection on red bayberry (Myrica rubra Sieb. & Zucc.) using fractal analysis and support vector machine. J. Food Eng..

[58] Hu M.-H., Dong Q.-L., Liu B.-L. (2016). Classification and characterization of blueberry mechanical damage with time evolution using reflectance, transmittance and interactance imaging spectroscopy. Comput. Electron. Agric..

[59] Qiu S., Wang J., Gao L. (2014). Discrimination and characterization of strawberry juice based on electronic nose and tongue: Comparison of different juice processing approaches by LDA, PLSR, RF, and SVM. J. Agric. Food Chem..

[60] Mireei S.A., Sadeghi M. (2013). Detecting bunch withering disorder in date fruit by near infrared spectroscopy. J. Food Eng..

[61] Hidalgo M.J., Fechner D.C., Marchevsky E.J., Pellerano R.G. (2016). Determining the geographical origin of Sechium edule fruits by multielement analysis and advanced chemometric techniques. Food Chem..

[62] Gaiad J.E., Hidalgo M.J., Villafañe R.N., Marchevsky E.J., Pellerano R.G. (2016). Tracing the geographical origin of Argentinean lemon juices based on trace element profiles using advanced chemometric techniques. Microchem. J..

[63] Canizo B.V., Escudero L.B., Pérez M.B., Pellerano R.G., Wuilloud R.G. (2018). Intra-regional classification of grape seeds produced in Mendoza province (Argentina) by multi-elemental analysis and chemometrics tools. Food Chem..

[64] Lubinska-Szczygieł M., Różańska A., Namieśnik J., Dymerski T., Shafreen R.B., Weisz M., Gorinstein S. (2018). Quality of limes juices based on the aroma and antioxidant properties. Food Control.

[65] Hernandez-Sanchez C., Luis G., Moreno I., Cameán A., González A.G., González-Weller D., Hardisson A. (2012). Differentiation of mangoes (*Magnifera indica* L.) conventional and organically cultivated according to their mineral content by using support vector machines. Talanta.

[66] Uçar A., Özalp R. (2017). Efficient android electronic nose design for recognition and perception of fruit odors using Kernel Extreme Learning Machines. Chemom. Intell. Lab. Syst..

[67] Guo Y., Ni Y., Kokot S. (2016). Evaluation of chemical components and properties of the jujube fruit using near infrared spectroscopy and chemometrics. Spectrochim. Acta A Mol. Biomol. Spectrosc..

[68] Yu K., Zhao Y., Li X., Shao Y., Zhu F., He Y. (2014). Identification of crack features in fresh jujube using Vis/NIR hyperspectral imaging combined with image processing. Comput. Electron. Agric..

[69] Munera S., Besada C., Aleixos N., Talens P., Salvador A., Sun D.W., Blasco J. (2017). Non-destructive assessment of the internal quality of intact persimmon using colour and vis/nir hyperspectral imaging. LWT.

[70] Sun Y., Gu X., Sun K., Hu H., Xu M., Wang Z., Pan L. (2017). Hyperspectral reflectance imaging combined with chemometrics and successive projections algorithm for chilling injury classification in peaches. LWT.

[71] Dutta M.K., Sengar N., Minhas N., Sarkar B., Goon A., Banerjee K. (2016). Image processing based classification of grapes after pesticide exposure. LWT—Food Sci. Technol..

[72] Cappellin L., Aprea E., Granitto P., Romano A., Gasperi F., Biasioli F. (2013). Multiclass methods in the analysis of metabolomic datasets: The example of raspberry cultivar volatile compounds detected by GC–MS and PTR-MS. Food Res. Int..

[73] Zheng W., Fu X., Ying Y. (2014). Spectroscopy-based food classification with extreme learning machine. Chemom. Intell. Lab. Syst..

[74] Gómez-Meire S., Campos C., Falqué E., Díaz F., Fdez-Riverola F. (2014). Assuring the authenticity of northwest Spain white wine varieties using machine learning techniques. Food Res. Int..

[75] Liu W., Liu C., Hu X., Yang J., Zheng L. (2016). Application of terahertz spectroscopy imaging for discrimination of transgenic rice seeds with chemometrics. Food Chem..

[76] Liu C., Liu W., Lu X., Chen W., Yang J., Zheng L. (2014). Nondestructive determination of transgenic Bacillus thuringiensis rice seeds (Oryza sativa L.) using multispectral imaging and chemometric methods. Food Chem..

[77] Yang P., Zhu Y., Yang X., Li J., Tang S., Hao Z., Lu Y. (2018). Evaluation of sample preparation methods for rice geographic origin classification using laser-induced breakdown spectroscopy. J. Cereal Sci..

[78] Feng X., Zhang Q., Cong P., Zhu Z. (2013). Preliminary study on classification of rice and detection of paraffin in the adulterated samples by Raman spectroscopy combined with multivariate analysis. Talanta.

[79] Barbosa R.M., de Paula E.S., Paulelli A.C., Moore A.F., Souza J.M.O., Batista B.L., Barbosa F. (2016). Recognition of organic rice samples based on trace elements and support vector machines. J. Food Compos. Anal..

[80] Lim D.K., Long N.P., Mo C., Dong Z., Cui L., Kim G., Kwon S.W. (2017). Combination of mass spectrometry-based targeted lipidomics and supervised machine learning algorithms in detecting adulterated admixtures of white rice. Food Res. Int..

[81] Siripatrawan U., Makino Y. (2015). Monitoring fungal growth on brown rice grains using rapid and non-destructive hyperspectral imaging. Int. J. Food Microbiol..

[82] Coïsson J.D., Arlorio M., Locatelli M., Garino C., Resta D., Sirtori E., Boschin G. (2011). The artificial intelligence-based chemometrical characterisation of genotype/chemotype of Lupinus albus and Lupinus angustifolius permits their identification and potentially their traceability. Food Chem..

[83] Marini F., Bucci R., Magrì A.L., Magrì A.D., Acquistucci R., Francisci R. (2008). Classification of 6 durum wheat cultivars from Sicily (Italy) using artificial neural networks. Chemom. Intell. Lab. Syst..

[84] Fernández Pierna J.A., Vermeulen P., Amand O., Tossens A., Dardenne P., Baeten V. (2012). NIR hyperspectral imaging spectroscopy and chemometrics for the detection of undesirable substances in food and feed. Chemom. Intell. Lab. Syst..

[85] Liu F., He Y. (2008). Classification of brands of instant noodles using Vis/NIR spectroscopy and chemometrics. Food Res. Int..

[86] Wakholi C., Kandpal L.M., Lee H., Bae H., Park E., Kim M.S., Cho B.K. (2018). Rapid assessment of corn seed viability using short wave infrared line-scan hyperspectral imaging and chemometrics. Sens. Actuators B Chem..

[87] Jia S., An D., Liu Z., Gu J., Li S., Zhang X., Yan Y. (2015). Variety identification method of coated maize seeds based on near-infrared spectroscopy and chemometrics. J. Cereal Sci..

[88] Jia S., Yang L., An D., Liu Z., Yan Y., Li S., Gu J. (2016). Feasibility of analyzing frost-damaged and non-viable maize kernels based on near infrared spectroscopy and chemometrics. J. Cereal Sci..

[89] Guo D., Zhu Q., Huang M., Guo Y., Qin J. (2017). Model updating for the classification of different varieties of maize seeds from different years by hyperspectral imaging coupled with a pre-labeling method. Comput. Electron. Agric..

[90] Ghasemi-Varnamkhasti M., Tohidi M., Mishra P., Izadi Z. (2018). Temperature modulation of electronic nose combined with multi-class support vector machine classification for identifying export caraway cultivars. Postharvest Biol. Technol..

[91] Rady A., Adedeji A. (2018). Assessing different processed meats for adulterants using visible-near-infrared spectroscopy. Meat. Sci..

[92] Schmutzler M., Beganovic A., Böhler G., Huck C.W. (2015). Methods for detection of pork adulteration in veal product based on FT-NIR spectroscopy for laboratory, industrial and on-site analysis. Food Control.

[93] Ropodi A.I., Panagou E.Z., Nychas G.-J.E. (2017). Multispectral imaging (MSI): A promising method for the detection of minced beef adulteration with horsemeat. Food Control.

[94] Matera J.A., Cruz A.G., Raices R.S.L., Silva M.C., Nogueira L.C., Quitério S.L., Júnior C.C. (2014). Discrimination of Brazilian artisanal and inspected pork sausages: Application of unsupervised, linear and non-linear supervised chemometric methods. Food Res. Int..

[95] Osorio M.T., Zumalacárregui J.M., Alaiz-Rodríguez R., Guzman-Martínez R., Engelsen S.B., Mateo J. (2009). Differentiation of perirenal and omental fat quality of suckling lambs according to the rearing system from Fourier transforms mid-infrared spectra using partial least squares and artificial neural networks analysis. Meat. Sci..

[96] Xu J.-L., Riccioli C., Sun D.-W. (2017). Comparison of hyperspectral imaging and computer vision for automatic differentiation of organically and conventionally farmed salmon. J. Food Eng..

[97] Martinez I., Standal I.B., Axelson D.E., Finstad B., Aursand M. (2009). Identification of the farm origin of salmon by fatty acid and HR 13C NMR profiling. Food Chem..

[98] Mohamadi Monavar H., Afseth N.K., Lozano J., Alimardani R., Omid M., Wold J.P. (2013). Determining quality of caviar from Caspian Sea based on Raman spectroscopy and using artificial neural networks. Talanta.

[99] Górska-Horczyczak E., Horczyczak M., Guzek D., Wojtasik-Kalinowska I., Wierzbicka A. (2017). Chromatographic fingerprints supported by artificial neural network for differentiation of fresh and frozen pork. Food Control.

[100] Li H., Sun X., Pan W., Kutsanedzie F., Zhao J., Chen Q. (2016). Feasibility study on nondestructively sensing meat’s freshness using light scattering imaging technique. Meat. Sci..

[101] Huang Q., Chen Q., Li H., Huang G., Ouyang Q., Zhao J. (2015). Non-destructively sensing pork’s freshness indicator using near infrared multispectral imaging technique. J. Food Eng..

[102] Xiao H., Liu M., Yuan H., Hong Q., Zhao J. (2013). Study on Detection and Classification of Tetracycline Residue in Duck Meat Using Synchronous Fluorescence Spectra and Support Vector Machine. J. Eng. Sci. Technol. Rev..

[103] Meisel S., Stöckel S., Rösch P., Popp J. (2014). Identification of meat-associated pathogens via Raman microspectroscopy. Food Microbiol..

[104] Li X., Kong W., Shi W., Shen Q. (2016). A combination of chemometrics methods and GC–MS for the classification of edible vegetable oils. Chemom. Intell. Lab. Syst..

[105] Luna A.S., da Silva A.P., Ferré J., Boqué R. (2013). Classification of edible oils and modeling of their physico-chemical properties by chemometric methods using mid-IR spectroscopy. Spectrochim. Acta A Mol. Biomol. Spectrosc..

[106] Jiménez-Carvelo A.M., Osorio M.T., Koidis A., González-Casado A., Cuadros-Rodríguez L. (2017). Chemometric classification and quantification of olive oil in blends with any edible vegetable oils using FTIR-ATR and Raman spectroscopy. LWT.

[107] Jimenez-Carvelo A.M., Pérez-Castaño E., González-Casado A., Cuadros-Rodríguez L. (2017). One input-class and two input-class classifications for differentiating olive oil from other edible vegetable oils by use of the normal-phase liquid chromatography fingerprint of the methyl-transesterified fraction. Food Chem..

[108] Zhou Y., Liu T., Li J. (2015). Rapid identification between edible oil and swill-cooked dirty oil by using a semi-supervised support vector machine based on graph and near-infrared spectroscopy. Chemom. Intell. Lab. Syst..

[109] Devos O., Downey G., Duponchel L. (2014). Simultaneous data pre-processing and SVM classification model selection based on a parallel genetic algorithm applied to spectroscopic data of olive oils. Food Chem..

[110] Sinues P.M., Alonso-Salces R.M., Zingaro L., Finiguerra A., Holland M.V., Guillou C., Cristoni S. (2012). Mass spectrometry fingerprinting coupled to National Institute of Standards and Technology Mass Spectral search algorithm for pattern recognition. Anal. Chim. Acta.

[111] Cajka T., Riddellova K., Klimankova E., Cerna M., Pudil F., Hajslova J. (2010). Traceability of olive oil based on volatiles pattern and multivariate analysis. Food Chem..

[112] Liu W., Liu C., Yu J., Zhang Y., Li J., Chen Y., Zheng L. (2018). Discrimination of geographical origin of extra virgin olive oils using terahertz spectroscopy combined with chemometrics. Food Chem..

[113] Torrecilla J.S., Cancilla J.C., Matute G., Díaz-Rodríguez P., Flores A.I. (2013). Self-organizing maps based on chaotic parameters to detect adulterations of extra virgin olive oil with inferior edible oils. J. Food Eng..

[114] Sanaeifar A., Jafari A., Golmakani M.T. (2018). Fusion of dielectric spectroscopy and computer vision for quality characterization of olive oil during storage. Comput. Electron. Agric..

[115] Zhang L.X., Shuai Q., Li P., Zhang Q., Ma F., Zhang W., Ding X. (2016). Ion mobility spectrometry fingerprints: A rapid detection technology for adulteration of sesame oil. Food Chem..

[116] Zhang L.X., Huang X., Li P., Na W., Jiang J., Mao J., Zhang Q. (2017). Multivariate adulteration detection for sesame oil. Chemom. Intell. Lab. Syst..

[117] Deng S., Xu Y., Li L., Li X., He Y. (2013). A feature-selection algorithm based on Support Vector Machine-Multiclass for hyperspectral visible spectral analysis. J. Food Eng..

[118] Luna A.S., da Silva A.P., Pinho J.S., Ferré J., Boqué R. (2013). Rapid characterization of transgenic and non-transgenic soybean oils by chemometric methods using NIR spectroscopy. Spectrochim. Acta A Mol. Biomol. Spectrosc..

[119] Lu Y., Du C., Yu C., Zhou J. (2014). Classifying rapeseed varieties using Fourier transform infrared photoacoustic spectroscopy (FTIR-PAS). Comput. Electron. Agric..

[120] Gorji-Chakespari A., Du C., Yu C., Zhou J. (2017). Classification of essential oil composition in Rosa damascena Mill. genotypes using an electronic nose. J. Appl. Res. Med. Aromat. Plants.

[121] Kuriakose S., Joe H. (2012). Qualitative and quantitative analysis in sandalwood oils using near infrared spectroscopy combined with chemometric techniques. Food Chem..

[122] Bougrini M., Tahri K., Haddi Z., El Bari N., Llobet E., Jaffrezic-Renault N., Bouchikhi B. (2014). Aging time and brand determination of pasteurized milk using a multisensor e-nose combined with a voltammetric e-tongue. Mater. Sci. Eng. C Mate.r Biol. Appl..

[123] Rodriguez-Bermudez R., López-Alonso M., Miranda M., Fouz R., Orjales I., Herrero-Latorre C. (2018). Chemometric authentication of the organic status of milk on the basis of trace element content. Food Chem..

[124] Zhang L.G., Zhang X., Ni L.J., Xue Z.B., Gu X., Huang S.X. (2014). Rapid identification of adulterated cow milk by non-linear pattern recognition methods based on near infrared spectroscopy. Food Chem..

[125] Lianou A., Malavazos C., Triantafyllou I., Nychas G.J.E., Panagou E.Z. (2017). Rapid Assessment of the Microbiological Quality of Pasteurized Vanilla Cream by Means of Fourier Transform Infrared Spectroscopy in Tandem with Support Vector Machine Analysis. Food Anal. Methods.

[126] Tohidi M., Ghasemi-Varnamkhasti M., Ghafarinia V., Mohtasebi S.S., Bonyadian M. (2018). Identification of trace amounts of detergent powder in raw milk using a customized low-cost artificial olfactory system: A novel method. Measurement.

[127] Ullah R., Khan S., Javaid S., Ali H., Bilal M., Saleem M. (2018). Raman spectroscopy combined with a support vector machine for differentiating between feeding male and female infants mother’s milk. Biomed. Opt. Express.

[128] Kowalski C.H., da Silva G.A., Godoy H.T., Poppi R.J., Augusto F. (2013). Application of Kohonen neural network for evaluation of the contamination of Brazilian breast milk with polychlorinated biphenyls. Talanta.

[129] Zhu X.R., Li S., Shan Y., Zhang Z., Li G., Su D., Liu F. (2010). Detection of adulterants such as sweeteners materials in honey using near-infrared spectroscopy and chemometrics. J. Food Eng..

[130] Herrero Latorre C., Crecente R.P., Martín S.G., García J.B. (2013). A fast chemometric procedure based on NIR data for authentication of honey with protected geographical indication. Food Chem..

[131] Stanimirova I., Üstün B., Cajka T., Riddelova K., Hajslova J., Buydens L.M.C., Walczak B. (2010). Tracing the geographical origin of honeys based on volatile compounds profiles assessment using pattern recognition techniques. Food Chem..

[132] Gan Z., Yang Y., Li J., Wen X., Zhu M., Jiang Y., Ni Y. (2016). Using sensor and spectral analysis to classify botanical origin and determine adulteration of raw honey. J. Food Eng..

[133] Batista B.L., Da Silva L.R.S., Rocha B.A., Rodrigues J.L., Berretta-Silva A.A., Bonates T.O., Barbosa F. (2012). Multi-element determination in Brazilian honey samples by inductively coupled plasma mass spectrometry and estimation of geographic origin with data mining techniques. Food Res. Int..

[134] El Alami El Hassani N., Tahri K., Llobet E., Bouchikhi B., Errachid A., Zine N., El Bari N. (2018). Emerging approach for analytical characterization and geographical classification of Moroccan and French honeys by means of a voltammetric electronic tongue. Food Chem..

[135] Barbosa R.M., Batista B.L., Varrique R.M., Coelho V.A., Campiglia A.D., Barbosa F. (2014). The use of advanced chemometric techniques and trace element levels for controlling the authenticity of organic coffee. Food Res. Int..

[136] Muñiz-Valencia R., Jurado J.M., Ceballos-Magaña S.G., Alcázar Á., Hernández-Díaz J. (2014). Characterization of Mexican coffee according to mineral contents by means of multilayer perceptrons artificial neural networks. J. Food Compos. Anal..

[137] Link J.V., Lemes A.L.G., Marquetti I., dos Santos Scholz M.B., Bona E. (2014). Geographical and genotypic classification of arabica coffee using Fourier transform infrared spectroscopy and radial-basis function networks. Chemom. Intell. Lab. Syst..

[138] Bona E., Marquetti I., Link J.V., Makimori G.Y.F., da Costa Arca V., Lemes A.L.G., Poppi R.J. (2017). Support vector machines in tandem with infrared spectroscopy for geographical classification of green arabica coffee. LWT—Food Sci. Technol..

[139] Palacios-Morillo A., Alcázar Á., de Pablos F., Jurado J.M. (2013). Differentiation of tea varieties using UV-Vis spectra and pattern recognition techniques. Spectrochim. Acta A Mol. Biomol. Spectrosc..

[140] Cai J.X., Wang Y.F., Xi X.G., Li H., Wei X.L. (2015). Using FTIR spectra and pattern recognition for discrimination of tea varieties. Int. J. Biol. Macromol..

[141] Cimpoiu C., Cristea V.M., Hosu A., Sandru M., Seserman L. (2011). Antioxidant activity prediction and classification of some teas using artificial neural networks. Food Chem..

[142] Wu D., Yang H., Chen X., He Y., Li X. (2008). Application of image texture for the sorting of tea categories using multi-spectral imaging technique and support vector machine. J. Food Eng..

[143] Xiong C., Liu C., Pan W., Ma F., Xiong C., Qi L., Zheng L. (2015). Non-destructive determination of total polyphenols content and classification of storage periods of Iron Buddha tea using multispectral imaging system. Food Chem..

[144] Wang S., Yang X., Zhang Y., Phillips P., Yang J., Yuan T.F. (2015). Identification of Green, Oolong and Black Teas in China via Wavelet Packet Entropy and Fuzzy Support Vector Machine. Entropy.

[145] Paneque P., Morales M.L., Burgos P., Ponce L., Callejón R.M. (2017). Elemental characterisation of Andalusian wine vinegars with protected designation of origin by ICP-OES and chemometric approach. Food Control.

[146] Rios-Reina R., Elcoroaristizabal S., Ocaña-González J.A., García-González D.L., Amigo J.M., Callejón R.M. (2017). Characterization and authentication of Spanish PDO wine vinegars using multidimensional fluorescence and chemometrics. Food Chem..

[147] Ji-yong S., Xiao-bo Z., Xiao-wei H., Jie-wen Z., Yanxiao L., Limin H., Jianchun Z. (2013). Rapid detecting total acid content and classifying different types of vinegar based on near infrared spectroscopy and least-squares support vector machine. Food Chem..

[148] Callejon R.M., Amigo J.M., Pairo E., Garmón S., Ocaña J.A., Morales M.L. (2012). Classification of Sherry vinegars by combining multidimensional fluorescence, parafac and different classification approaches. Talanta.

[149] Jurado J.M., Alcázar Á., Palacios-Morillo A., de Pablos F. (2012). Classification of Spanish DO white wines according to their elemental profile by means of support vector machines. Food Chem..

[150] Selih V.S., Sala M., Drgan V. (2014). Multi-element analysis of wines by ICP-MS and ICP-OES and their classification according to geographical origin in Slovenia. Food Chem..

[151] Cetó X., González-Calabuig A., Capdevila J., Puig-Pujol A., Del Valle M. (2015). Instrumental measurement of wine sensory descriptors using a voltammetric electronic tongue. Sens. Actuators B Chem..

[152] Debska B., Guzowska-Swider B. (2011). Application of artificial neural network in food classification. Anal Chim. Acta.

[153] Silva G.A., Augusto F., Poppi R.J. (2008). Exploratory analysis of the volatile profile of beers by HS–SPME–GC. Food Chem..

[154] Alcázar Á., Jurado J.M., Palacios-Morillo A., de Pablos F., Martín M.J. (2012). Recognition of the geographical origin of beer based on support vector machines applied to chemical descriptors. Food Control.

[155] Iglesias Rodriguez R., Delgado M.F., García J.B., Crecente R.M.P., Martín S.G., Latorre C.H. (2010). Comparison of several chemometric techniques for the classification of orujo distillate alcoholic samples from Galicia (northwest Spain) according to their certified brand of origin. Anal. Bioanal. Chem..

[156] Contreras U., Barbosa-García O., Pichardo-Molina J.L., Ramos-Ortíz G., Maldonado J.L., Meneses-Nava M.A., López-de-Alba P.L. (2010). Screening method for identification of adulterate and fake tequilas by using UV–VIS spectroscopy and chemometrics. Food Res. Int..

[157] Pérez-Caballero G., Andrade J.M., Olmos P., Molina Y., Jiménez I., Durán J.J., Miguel-Cruz F. (2017). Authentication of tequilas using pattern recognition and supervised classification. TrAC Trends Anal. Chem..

[158] Rodrigues B.U., Soares A.D.S., Costa R.M.D., Van Baalen J., Salvini R.L., Silva F.A.D., Federson F.M. (2016). A feasibility cachaca type recognition using computer vision and pattern recognition. Comput. Electron. Agric..

[159] Liu Y., Li L., Xiao Y.Q., Yao J.Q., Li P.Y., Yu D.R., Ma Y.L. (2016). Global metabolite profiling and diagnostic ion filtering strategy by LC-QTOF MS for rapid identification of raw and processed pieces of *Rheum palmatum* L.. Food Chem..

[160] Ni Y., Song R., Kokot S. (2012). Discrimination of Radix Isatidis and Rhizoma et Radix Baphicacanthis Cusia samples by near infrared spectroscopy with the aid of chemometrics. Spectrochim. Acta A Mol. Biomol. Spectrosc..

[161] Teye E., Huang X.Y., Lei W., Dai H. (2014). Feasibility study on the use of Fourier transform near-infrared spectroscopy together with chemometrics to discriminate and quantify adulteration in cocoa beans. Food Res. Int..

[162] Vargas Jentzsch P., Ciobotă V., Salinas W., Kampe B., Aponte P.M., Rösch P., Ramos L.A. (2016). Distinction of Ecuadorian varieties of fermented cocoa beans using Raman spectroscopy. Food Chem..

[163] Grošelj N., van der Veer G., Tušar M., Vračko M., Novič M. (2010). Verification of the geological origin of bottled mineral water using artificial neural networks. Food Chem..

[164] Marcelo M.C.A., Martins C.A., Pozebon D., Dressler V.L., Ferrão M.F. (2014). Classification of yerba mate (Ilex paraguariensis) according to the country of origin based on element concentrations. Microchem. J..

[165] Zhuang H., Ni Y., Kokot S. (2014). Combining HPLC–DAD and ICP-MS data for improved analysis of complex samples: Classification of the root samples from Cortex moutan. Chemom. Intell. Lab. Syst..

[166] Li C., Yang S.C., Guo Q.S., Zheng K.Y., Shi Y.F., Xiao X.F., Long G.Q. (2014). Determining the geographical origin of the medicinal plant Marsdenia tenacissima with multi-element analysis and data mining techniques. Chemom. Intell. Lab. Syst..

[167] Kwon Y.K., Bong Y.S., Lee K.S., Hwang G.S. (2014). An integrated analysis for determining the geographical origin of medicinal herbs using ICP-AES/ICP-MS and (1)H NMR analysis. Food Chem..

[168] Liu Z., Xu H. (2014). Kernel Parameter Selection for Support Vector Machine Classification. J. Algorithms Comput. Technol..

[169] Luts J., Ojeda F., Van de Plas R., De Moor B., Van Huffel S., Suykens J.A. (2010). A tutorial on support vector machine-based methods for classification problems in chemometrics. Anal. Chim. Acta.

[170] Wei Z.B., Wang J. (2013). The evaluation of sugar content and firmness of non-climacteric pears based on voltammetric electronic tongue. J. Food Eng..

[171] Batista G.E.A.P.A., Prati R.C., Monard M.C. (2005). Balancing Strategies and Class Overlapping.

[172] Lin M., Tang K., Yao X. (2013). Dynamic Sampling Approach to Training Neural Networks for Multiclass Imbalance Classification. IEEE Trans. Neural Netw. Learn. Syst..

[173] Byrd R.H., Chin G.M., Nocedal J., Wu Y. (2012). Sample size selection in optimization methods for machine learning. Math. Program..

[174] Wang S., Yao X. (2012). Multiclass Imbalance Problems: Analysis and Potential Solutions. IEEE Trans. Syst. Man Cybern. Part B.

[175] Hall M.A., Smith L.A. (1998). Practical Feature Subset Selection for Machine Learning.

[176] Gaspar P., Carbonell J., Oliveira J.L. (2012). On the parameter optimization of Support Vector Machines for binary classification. J. Integr. Bioinform..

[177] Martens H., Naes T. (1984). Multivariate Calibration.

[178] Liu C., Liu W., Chen W., Yang J., Zheng L. (2015). Feasibility in multispectral imaging for predicting the content of bioactive compounds in intact tomato fruit. Food Chem..

[179] Niu X., Zhao Z., Jia K., Li X. (2012). A feasibility study on quantitative analysis of glucose and fructose in lotus root powder by FT-NIR spectroscopy and chemometrics. Food Chem..

[180] Liu C., Liu W., Lu X., Chen W., Yang J., Zheng L. (2016). Potential of multispectral imaging for real-time determination of colour change and moisture distribution in carrot slices during hot air dehydration. Food Chem..

[181] Ni Y., Xiao W., Kokot S. (2009). Application of chemometrics methods for the simultaneous kinetic spectrophotometric determination of aminocarb and carbaryl in vegetable and water samples. J. Hazard. Mater..

[182] Siripatrawan U., Harte B.R. (2015). Data visualization of Salmonella Typhimurium contamination in packaged fresh alfalfa sprouts using a Kohonen network. Talanta.

[183] Cerit I., Yildirim A., UCAR;MK;Demirkol A., Cosansu S., Demirkol O. (2017). Estimation of antioxidant activity of foods using artificial neural networks. J. Food Nutr. Res..

[184] Jafari S.M., Ghanbari V., Ganje M., Dehnad D. (2016). Modeling the Drying Kinetics of Green Bell Pepper in a Heat Pump Assisted Fluidized Bed Dryer. J. Food Q..

[185] Liu C., Hao G., Su M., Chen Y., Zheng L. (2017). Potential of multispectral imaging combined with chemometric methods for rapid detection of sucrose adulteration in tomato paste. J. Food Eng..

[186] Siripatrawan U., Makino Y., Kawagoe Y., Oshita S. (2011). Rapid detection of Escherichia coli contamination in packaged fresh spinach using hyperspectral imaging. Talanta.

[187] Li J.B., Huang W., Zhao C., Zhang B. (2013). A comparative study for the quantitative determination of soluble solids content, pH and firmness of pears by Vis/NIR spectroscopy. J. Food Eng..

[188] Das M., Akpinar E.K. (2018). Investigation of Pear Drying Performance by Different Methods and Regression of Convective Heat Transfer Coefficient with Support Vector Machine. Appl. Sci..

[189] Conesa C., Ibanez Civera J., Seguí L., Fito P., Laguarda-Miró N. (2016). An Electrochemical Impedance Spectroscopy System for Monitoring Pineapple Waste Saccharification. Sensors.

[190] Guo Z.M., Huang W., Peng Y., Chen Q., Ouyang Q., Zhao J. (2016). Color compensation and comparison of shortwave near infrared and long wave near infrared spectroscopy for determination of soluble solids content of ‘Fuji’ apple. Postharvest Biol. Technol..

[191] Cao F., Wu D., He Y. (2010). Soluble solids content and pH prediction and varieties discrimination of grapes based on visible-near infrared spectroscopy. Comput. Electron. Agric..

[192] Malegori C., Marques E.J.N., de Freitas S.T., Pimentel M.F., Pasquini C., Casiraghi E. (2017). Comparing the analytical performances of Micro-NIR and Ft-NIR spectrometers in the evaluation of acerola fruit quality, using PLS and SVM regression algorithms. Talanta.

[193] Sanaeifar A., Mohtasebi S.S., Ghasemi-Varnamkhasti M., Ahmadi H. (2016). Application of MOS based electronic nose for the prediction of banana quality properties. Measurement.

[194] Zhu N., Lin M., Nie Y., Wu D., Chen K. (2016). Study on the quantitative measurement of firmness distribution maps at the pixel level inside peach pulp. Comput. Electron. Agric..

[195] Xue J.X., Zhang S., Sun H., Zhou J. (2013). Study of Malus Asiatica Nakai’s firmness during different shelf lives based on visible/near-infrared spectroscopy. Math. Comput. Model..

[196] Hu M.H., Dong Q.L., Liu B.L., Opara U.L. (2016). Prediction of mechanical properties of blueberry using hyperspectral interactance imaging. Postharvest Biol. Technol..

[197] Cortes V., Rodriguez A., Blasco J., Rey B., Besada C., Cubero S., Aleixos N. (2017). Prediction of the level of astringency in persimmon using visible and near-infrared spectroscopy. J. Food Eng..

[198] Yang Y.C., Sun D.W., Wang N.N. (2015). Rapid detection of browning levels of lychee pericarp as affected by moisture contents using hyperspectral imaging. Comput. Electron. Agric..

[199] Huang L.X., Zhou Y., Meng L., Wu D., He Y. (2017). Comparison of different CCD detectors and chemometrics for predicting total anthocyanin content and antioxidant activity of mulberry fruit using visible and near infrared hyperspectral imaging technique. Food Chem..

[200] Qiu S.S., Wang J. (2017). The prediction of food additives in the fruit juice based on electronic nose with chemometrics. Food Chem..

[201] Marini F. (2009). Artificial neural networks in foodstuff analyses: Trends and perspectives A review. Anal. Chim. Acta.

[202] Mariani N.C.T., da Costa R.C., de Lima K.M.G., Nardini V., Júnior L.C.C., de Almeida Teixeira G.H. (2014). Predicting soluble solid content in intact jaboticaba [Myrciaria jaboticaba (Vell.) O. Berg fruit using near-infrared spectroscopy and chemometrics. Food Chem..

[203] Peng J.T., Li L.Q., Tang Y.Y. (2013). Combination of activation functions in extreme learning machines for multivariate calibration. Chemom. Intell. Lab. Syst..

[204] Abbasi-Tarighat M., Shahbazi E., Niknam K. (2013). Simultaneous determination of Mn^2+^ and Fe^3+^ as 4,4 ‘[(4-cholorophenyl)methylene bis(3-methyl-1-phenyl-1H-pyrazol-5-ol) complexes in some foods, vegetable and water samples by artificial neural networks. Food Chem..

[205] Funsueb S., Krongchai C., Mahatheeranont S., Kittiwachana S. (2016). Prediction of 2-acetyl-1-pyrroline content in grains of Thai Jasmine rice based on planting condition, plant growth and yield component data using chemometrics. Chemom. Intell. Lab. Syst..

[206] Shao Y.N., Cen Y., He Y., Liu F. (2011). Infrared spectroscopy and chemometrics for the starch and protein prediction in irradiated rice. Food Chem..

[207] Das B., Sahoo R.N., Pargal S., Krishna G., Verma R., Chinnusamy V., Swain P. (2018). Quantitative monitoring of sucrose, reducing sugar and total sugar dynamics for phenotyping of water-deficit stress tolerance in rice through spectroscopy and chemometrics. Spectrochim. Acta Part A Mol. Biomol. Spectrosc..

[208] Sahoo R.N., Lu S., Liao Y., Zhang Z. (2017). Simultaneous determination of amino acid mixtures in cereal by using terahertz time domain spectroscopy and chemometrics. Chemom. Intell. Lab. Syst..

[209] Coen T., Saeys W., Ramon H., De Baerdemaeker J. (2006). Optimizing the tuning parameters of least squares support vector machines regression for NIR spectra. J. Chemom..

[210] Thissen U., Pepers M., Üstün B., Melssen W.J., Buydens L.M.C. (2004). Comparing support vector machines to PLS for spectral regression applications. Chemom. Intell. Lab. Syst..

[211] Fu H.Y., Li H.D., Xu L., Yin Q.B., Yang T.M., Ni C., She Y.B. (2017). Detection of unexpected frauds: Screening and quantification of maleic acid in cassava starch by Fourier transform near-infrared spectroscopy. Food Chem..

[212] Li J.T., Zhu S., Jiang S., Wang J. (2017). Prediction of egg storage time and yolk index based on electronic nose combined with chemometric methods. LWT-Food Sci. Technol..

[213] Papadopoulos V.D., Beligiannis G.N., Hela D.G. (2011). Combining experimental design and artificial neural networks for the determination of chlorinated compounds in fish using matrix solid-phase dispersion. Appl. Soft. Comput..

[214] Xu J., Riccioli C., Sun D.-W. (2016). Development of an alternative technique for rapid and accurate determination of fish caloric density based on hyperspectral imaging. J. Food Eng..

[215] Cheng J.-H., Sun D.W., Zeng X.A., Pu H.B. (2014). Non-destructive and rapid determination of TVB-N content for freshness evaluation of grass carp (Ctenopharyngodon idella) by hyperspectral imaging. Innov. Food Sci. Emerg. Technol..

[216] Papadopoulou O., Panagou E.Z., Mohareb F.R., Nychas G.J.E. (2013). Sensory and microbiological quality assessment of beef fillets using a portableelectronic nose in tandem with support vector machine analysis. Food Res. Int..

[217] Prevolnik M., Čandek-Potokar M., Novič M., Škorjanc D. (2009). An attempt to predict pork drip loss from pH and colour measurements or near infrared spectra using artificial neural networks. Meat. Sci..

[218] Yang D.Z., Li H., Cao C., Chen F., Zhou Y., Xiu Z. (2014). Analysis of the Oil Content of Rapeseed Using Artificial Neural Networks Based on Near Infrared Spectral Data. J. Spectrosc..

[219] Cabrera A.C., Prieto J.M. (2010). Application of artificial neural networks to the prediction of the antioxidant activity of essential oils in two experimental in vitro models. Food Chem..

[220] Dong W., Zhang Y., Zhang B., Wang X. (2012). Quantitative analysis of adulteration of extra virgin olive oil using Raman spectroscopy improved by Bayesian framework least squares support vector machines. Anal. Methods.

[221] Zhang C., Kong W., Liu F., He Y. (2016). Measurement of aspartic acid in oilseed rape leaves under herbicide stress using near infrared spectroscopy and chemometrics. Heliyon.

[222] Riahi S., Pourbasheer E., Ganjali M.R., Norouzi P. (2009). Investigation of different linear and nonlinear chemometric methods for modeling of retention index of essential oil components: Concerns to support vector machine. J. Hazard. Mater..

[223] Bassbasi M., Platikanov S., Tauler R., Oussama A. (2014). FTIR-ATR determination of solid non fat (SNF) in raw milk using PLS and SVM chemometric methods. Food Chem..

[224] Wei Z.B., Zhang W., Wang Y., Wang J. (2017). Monitoring the fermentation, post-ripeness and storage processes of set yogurt using voltammetric electronic tongue. J. Food Eng..

[225] Da Rocha R.A., Paiva I.M., Anjos V., Furtado M.A.M., Bell M.J.V. (2015). Quantification of whey in fluid milk using confocal Raman microscopy and artificial neural network. J. Dairy Sci..

[226] Altieri G., Genovese F., Admane N., Di Renzo G.C. (2016). On-line measure of donkey’s milk properties by near infrared spectrometry. Lwt-Food Sci. Technol..

[227] Wu D., He Y., Feng S., Sun D.W. (2008). Study on infrared spectroscopy technique for fast measurement of protein content in milk powder based on LS-SVM. J. Food Eng..

[228] Balabin R.M., Smirnov S.V. (2011). Melamine detection by mid- and near-infrared (MIR/NIR) spectroscopy: A quick and sensitive method for dairy products analysis including liquid milk, infant formula, and milk powder. Talanta.

[229] Tan C., Qin X., Li M. (2008). An ensemble method based on a self-organizing map for near-infrared spectral calibration of complex beverage samples. Anal. Bioanal. Chem..

[230] Wu D., Chen J., Lu B., Xiong L., He Y., Zhang Y. (2012). Application of near infrared spectroscopy for the rapid determination of antioxidant activity of bamboo leaf extract. Food Chem..

[231] Ouyang Q., Zhao J.W., Chen Q.S. (2014). Instrumental intelligent test of food sensory quality as mimic of human panel test combining multiple cross-perception sensors and data fusion. Anal. Chim. Acta.

[232] Ge H.Y., Jiang Y., Lian F., Zhang Y., Xia S. (2016). Quantitative determination of aflatoxin B1 concentration in acetonitrile by chemometric methods using terahertz spectroscopy. Food Chem..

[233] Rodriguez S.D., Monge M.E., Olivieri A.C., Negri R.M., Bernik D.L. (2010). Time dependence of the aroma pattern emitted by an encapsulated essence studied by means of electronic noses and chemometric analysis. Food Res. Int..

[234] Liu F., He Y. (2009). Application of successive projections algorithm for variable selection to determine organic acids of plum vinegar. Food Chem..

[235] Wu Z.Z., Xu E., Long J., Pan X., Xu X., Jin Z., Jiao A. (2016). Comparison between ATR-IR, Raman, concatenated ATR-IR and Raman spectroscopy for the determination of total antioxidant capacity and total phenolic content of Chinese rice wine. Food Chem..

[236] Liu F., He Y., Wang L. (2008). Determination of effective wavelengths for discrimination of fruit vinegars using near infrared spectroscopy and multivariate analysis. Anal. Chim. Acta.

[237] Rasouli Z., Ghavami R. (2016). Investigating the discrimination potential of linear and nonlinear spectral multivariate calibrations for analysis of phenolic compounds in their binary and ternary mixtures and calculation pKa values. Spectrochim. Acta A Mol. Biomol. Spectrosc..

[238] Ramirez-Morales I., Rivero D., Fernández-Blanco E., Pazos A. (2016). Optimization of NIR calibration models for multiple processes in the sugar industry. Chemom. Intell. Lab. Syst..

[239] Cheng P.Y., Fan W.L., Xu Y. (2013). Quality grade discrimination of Chinese strong aroma type liquors using mass spectrometry and multivariate analysis. Food Res. Int..

[240] Snedecor G.W., Cochran W.G. (1967). Statistical Methods.

[241] Akaike H., Parzen E., Tanabe K., Kitagawa G. (1998). Information Theory and an Extension of the Maximum Likelihood Principle. Selected Papers of Hirotugu Akaike.

[242] Snipes M., Taylor D.C. (2014). Model selection and Akaike Information Criteria: An example from wine ratings and prices. Wine Econ. Policy.

[243] Frank J., Focardi S.M., Rachev S.T., Arshanapalli B.G. (2014). The Basics of Financial Econometrics: Tools, Concepts, and Asset Management Applications.

[244] Ng M., Wilcox R.E. (2012). Bootstrap methods for comparing independent regression slopes. Br. J. Math. Stat. Psychol..

[245] Westad F., Marini F. (2015). Validation of Chemometric Models—A tutorial. Anal. Chim. Acta.

[246] Anscombe F.J. (1973). Graphs in Statistical Analysis. Am. Stat..

[247] Defernez M., Kemsley E.K. (1997). The use and misuse of chemometrics for treating classification problems. TrAC Trends Anal. Chem..

[248] De Bièvre P. (1997). ACQUAL welcomes Japan. Accredit. Q. Assur..

[249] Kreiss J., Paparoditis E. (2011). Bootstrap methods for dependent data: A review. J. Korean Stat. Soc..

[250] Datta J., Ghosh J.K. (2014). Bootstrap—An exploration. Stat. Methodol..

[251] Martens H., Martens M. (2000). Modified Jack-knife estimation of parameter uncertainty in bilinear modelling by partial least squares regression (PLSR). Food Q. Pref..

